# The RNase H-like superfamily: new members, comparative structural analysis and evolutionary classification

**DOI:** 10.1093/nar/gkt1414

**Published:** 2014-01-23

**Authors:** Karolina A. Majorek, Stanislaw Dunin-Horkawicz, Kamil Steczkiewicz, Anna Muszewska, Marcin Nowotny, Krzysztof Ginalski, Janusz M. Bujnicki

**Affiliations:** ^1^Laboratory of Bioinformatics and Protein Engineering, International Institute of Molecular and Cell Biology, ul. Ks. Trojdena 4, PL-02-109 Warsaw, Poland, ^2^Department of Molecular Physiology and Biological Physics, University of Virginia, 1340 Jefferson Park Avenue, Charlottesville, VA USA-22908, USA, ^3^Bioinformatics Laboratory, Institute of Molecular Biology and Biotechnology, Adam Mickiewicz University, Umultowska 89, PL-61-614 Poznan, Poland, ^4^Laboratory of Bioinformatics and Systems Biology, Centre of New Technologies, University of Warsaw, Zwirki i Wigury 93, PL-02-089 Warsaw, Poland, ^5^Institute of Biochemistry and Biophysics PAS, Pawinskiego 5A, PL-02-106 Warsaw, Poland and ^6^Laboratory of Protein Structure, International Institute of Molecular and Cell Biology, ul. Ks. Trojdena 4, PL-02-109 Warsaw, Poland

## Abstract

Ribonuclease H-like (RNHL) superfamily, also called the retroviral integrase superfamily, groups together numerous enzymes involved in nucleic acid metabolism and implicated in many biological processes, including replication, homologous recombination, DNA repair, transposition and RNA interference. The RNHL superfamily proteins show extensive divergence of sequences and structures. We conducted database searches to identify members of the RNHL superfamily (including those previously unknown), yielding >60 000 unique domain sequences. Our analysis led to the identification of new RNHL superfamily members, such as RRXRR (PF14239), DUF460 (PF04312, COG2433), DUF3010 (PF11215), DUF429 (PF04250 and COG2410, COG4328, COG4923), DUF1092 (PF06485), COG5558, OrfB_IS605 (PF01385, COG0675) and Peptidase_A17 (PF05380). Based on the clustering analysis we grouped all identified RNHL domain sequences into 152 families. Phylogenetic studies revealed relationships between these families, and suggested a possible history of the evolution of RNHL fold and its active site. Our results revealed clear division of the RNHL superfamily into exonucleases and endonucleases. Structural analyses of features characteristic for particular groups revealed a correlation between the orientation of the C-terminal helix with the exonuclease/endonuclease function and the architecture of the active site. Our analysis provides a comprehensive picture of sequence-structure-function relationships in the RNHL superfamily that may guide functional studies of the previously uncharacterized protein families.

## INTRODUCTION

The ribonuclease H-like (RNHL) superfamily is a large group of evolutionarily related, but strongly diverged, proteins with different functions. Ribonuclease (RNase) H from *E. coli* was the first protein of the superfamily for which the 3D structure was determined, revealing a new architecture of the polypeptide chain, subsequently called the RNase H fold (1,2). Similar spatial architecture of the catalytic domain was later identified in other enzymes involved in nucleic acid metabolism, including retroviral integrases and DNA transposases (3), Holliday junction resolvases (HJRs) (4), Piwi/Argonaute nucleases (5,6), numerous exonucleases (7) and Prp8: the largest and most highly conserved spliceosomal protein considered to be a master regulator of the spliceosome (8). RNase H-like enzymes are involved in numerous fundamental processes, including DNA replication and repair, homologous recombination, transposition and RNA interference.

RNHL superfamily proteins, despite extensive sequence and function diversity, show significant similarity of the global 3D fold, the architecture of the catalytic core and the catalytic mechanism. The RNase H-like fold has been shown to be one of the evolutionarily oldest protein folds (9). The main element of the RNase H-like catalytic core is a β-sheet comprising five β-strands, ordered 32 145, where the β-strand 2 is antiparallel to the other β-strands. On both sides the central β-sheet is flanked by α-helices, the number of which differs between related enzymes. RNHL superfamily members also share the position and type of the active site residues, which typically include aspartic acid, glutamic acid and in some cases histidine. Positions of the two key aspartate residues are most strongly conserved, while the position and identity of the remaining catalytic amino acid residues differ to some extent between members of particular families. Negatively charged side chains in the active sites of the RNase H-like enzymes are involved, directly or through the water molecule, in coordination of divalent metal ions. It has been shown that RNase H-like enzymes use a two ion-dependent mechanism of catalysis (2,10–13). Under physiological conditions, the preferred ion is Mg^2+^, but Mn^2+^ also supports catalysis, while Ca^2+^ inhibits the cleavage (14).

Many enzymes have been classified as RNHL superfamily members, including RNases and deoxyribonucleases, exo- and endonucleases, proteins that fulfill numerous functions in Eukaryota, Prokaryota, Archaea and viruses. For many of these proteins, 3D structures have been solved, e.g. the SCOP database, as of August 2013 lists 14 protein families with experimentally determined structures, classified as the members of the RNHL superfamily. However, for most members of the RNHL superfamily identified to date, the structural information is missing and their evolutionary origin is typically unknown. In addition, discoveries of unexpected RNase H-like structures in various proteins, such as Prp8p, suggest that further RNHL superfamily members remain to be discovered.

In this work, we carried out a search for all proteins, which, based on their structural and catalytic properties, can be incorporated into the RNHL superfamily. We have subsequently analyzed sequence-structure-function relationships and developed a classification scheme for previously known and new members of the RNHL superfamily, which will greatly facilitate computational annotations of proteins and domains and the planning of experiments to reveal their biochemical and cellular functions.

## MATERIALS AND METHODS

### Sequence and structure database searches and clustering analysis

Structures of known members of the RNHL superfamily were selected from the SCOP database (1.75 release) (15). For each family at least one representative structure was selected, giving preference to structures determined by radiograph crystallography with the highest resolution. Among 14 SCOP families, we selected 17 representative structures (List 1 in Supplementary File S1). These representatives were used as queries to search the Protein Data Bank (PDB) with DALI (16) to identify proteins with a similar topology, including those not yet assigned to the RNHL superfamily in the SCOP database. We conducted analysis of pairwise structural similarities between the previously known members of the RNase H superfamily using the DaliLite server. Based on the Z-scores assigned to each compared pair of known homologs, we attempted to determine the Z-score value threshold that would allow us to automatically include newly identified similar structures in further steps of the analysis. Initially, new structures identified by DALI with Z score ≥4 were automatically included in further steps of the analysis, while structures with Z scores <4 were checked for similarity of their function and analyzed visually to confirm their similarity to RNase H fold. In some cases, for the structures of proteins with unknown function, false positives or false negatives could be generated, due to the relatively high similarity of the RNase H-like superfamily members to the Actin-like ATPase domain that shares the same fold.

Amino acid sequences corresponding to the collected structures of RNHL superfamily members were clustered using CLANS (17), so that proteins showing sequence similarity could be grouped into families. CLANS uses the *P*-values of highly scoring segment pairs obtained from an N × N BLAST search, to compute attractive and repulsive forces between each sequence pair in a user-defined data set. A 2D or 3D representation of sequence families is achieved by randomly seeding the sequences in the arbitrary distance space, then moving them within this environment according to the force vectors resulting from all pairwise interactions, and repeating the process until convergence, based on the Fruchterman–Reingold graph layout algorithm (17). The *P*-value threshold of 1e-12 was used for the clustering; the clusters obtained were robust, as clustering with a more permissive threshold of 1e-2 has yielded qualitatively similar results (data not shown). Sequence similarity-based clustering was used three times in this analysis, first to group the sequences representing proteins with known structures, second to group representatives of Pfam, COG and KOG databases and third to group all identified RNHL-like domains.

Based on the results of clustering performed with CLANS, we selected representative sequences for each cluster. We gave preference to proteins with known function and/or classified as members of the RNase H-like superfamily in the SCOP database (e.g. functionally uncharacterized structures and not in SCOP had the lowest priority to become representative members of a family). Orphan sequences that did not connect to any cluster were also included in the representative sequences set, after visual validation of the corresponding structures, to confirm their similarity to RNase H fold. As a result, we obtained a set of 51 RNHL members with known structure (List 2 in Supplementary File S1), which we used as main queries in our searches and in further comparative analyses.

The sequences of the representative members of the RNHL superfamily (List 2 in Supplementary File S1) were used as queries to search a database of profile-HMMs corresponding to alignments of protein families included in the COG, KOG (18) and Pfam (19) databases, using the HHpred server (20) with default parameters. HHpred builds a multiple sequence alignment for the query sequence by multiple iterations of searches of the nonredundant (nr) sequence database at the NCBI, and generates a profile-HMM that includes information about both sequence and predicted secondary structure. The query profile-HMM is then compared with precalculated profile-HMMs in the selected database with the HHsearch method for HMM–HMM comparison (21). Finally, the set of RNHL families and structures identified by this search was used for further comprehensive searches for novel, most diverged members of RNHL superfamily, using highly sensitive, distant homology detection method Meta-BASIC (22). Meta-BASIC is a meta-profile alignment method capable of finding distant similarity between proteins through a comparison of sequence profiles, generated with PSI-BLAST using NCBI nr protein sequence database derivative (NR70), and enriched by predicted secondary structures. Identification of novel RNHL families was carried out using our database of precalculated Meta-BASIC connections between all Pfam, COG and KOG families and proteins of known structure (representatives from PDB filtered at 90% of sequence identity). The search strategy was based on the transitivity concept, where each newly identified Pfam, COG and KOG family was used for further Meta-BASIC searches until no new additional hits were found. Moreover, the high divergence of some RNHL families that is likely reflected as lower than confidence threshold (<40) Meta-BASIC scores was also considered. Specifically, in addition to high scoring (>40) Meta-BASIC hits, hits below threshold (with scores >20) were also analyzed to identify correct predictions placed among incorrect ones. These potentially novel superfamily members were subjected to further extensive analyses including fold recognition with 3D-Jury (23) to confirm initial predictions. The final selection of correct but nontrivial assignments was based on the consistency of a predicted secondary structure pattern with that of RNase H fold, general conservation of critical hydrophobic positions and, for potentially active enzymes, presence of active site residues.

Sequences corresponding to multiple sequence alignments from the COG, KOG and Pfam entries predicted to be homologous to any of the RNHL queries were downloaded and clustered using CLANS (17). Sequences from each of the clusters were aligned using MUSCLE (24). Representative sequences (one per cluster) were selected and used as queries in the final series of PSI-BLAST searches of the nr database. For each sequence, the search was run with the expectation (e) value threshold for the retrieval of related sequences set to 1e^−3^, and we retrieved all sequences reported with e-value <1e^−3^ (over a total number of 170 000 sequences).

**Figure 1.**
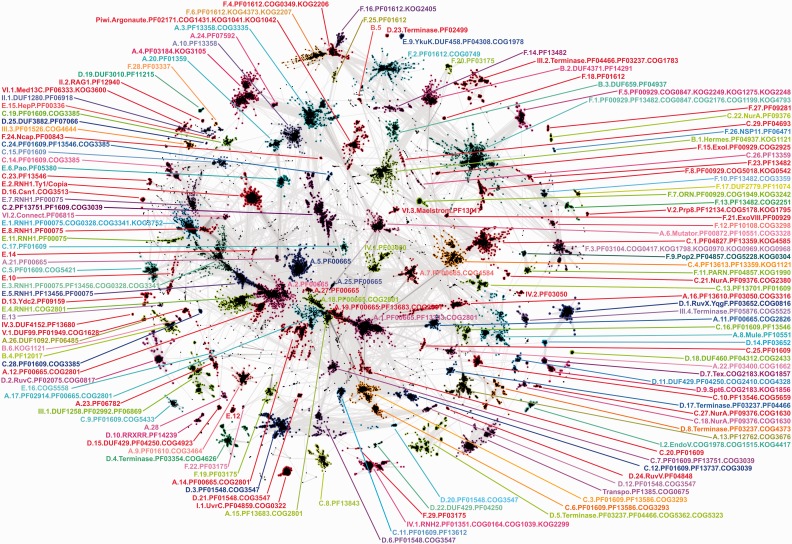
Two-dimensional projection of the CLANS clustering results obtained for the sequences of the RNHL domains of the superfamily proteins. Sequences are indicated by dots. Lines indicate sequence similarity detectable with BLAST and are colored by a spectrum of shades of gray according to the BLAST *P*-value. To facilitate distinction of separate clusters they are indicated with different colors matching the colors of their labels. Clusters were labeled with the clade they grouped into in the evolutionary analysis (indicated with letters A–F and Roman numerals I–VI; see section Phylogenetic analysis). For each clade the clusters were numbered based on their size in descending order (e.g. cluster A.1. will comprise more sequences than the cluster A.8.). The number is followed by a common name of the most distinguished member of the cluster and identifiers of Pfam, COG and KOG families, members of which were found in the cluster.

Based on the alignments obtained in the previous step, we mapped the position of the RNHL domains in the identified full-length protein sequences. The extracted sequences of the RNHL domains were used in further steps of the analysis. To reduce the size of the resulting data set of RNHL domains, we clustered the sequences using CD-HIT (25) with 80% identity threshold; thus, the sequences with ≥80% identity to the representative sequences were removed. As a result, we obtained a nr set of 61 923 RNHL domain sequences (Supplementary File S2). To divide the RNHL superfamily into families, the final set of domain sequences was clustered using CLANS. Owing to a large number of sequences with different levels of similarity, to produce the qualitatively best results, the clusters were defined by visual analysis of the clustering diagram using different *P*-value thresholds, starting with 1e-9 for identification of the most diverged sequences. Subsequently, more stringent threshold values were introduced to elucidate subgroups within more connected cluster groups. Based on the results of the clustering, 152 families were defined (the division is indicated in Figure 1 and in Supplementary File S2). For further analysis, representative sequences were selected for each of 152 clusters (Supplementary File S3). For clusters that contained at least one RNHL domain with known structure, one structure per cluster was selected as a representative, giving 41 structurally characterized representatives in total.

### Structure-based sequence alignment

Because of significant sequence divergence of RNHL superfamily members, the sequence alignment was prepared based on the superposition of their conserved structures. The 41 representative structures of RNHL superfamily enzymes (Supplementary File S1) were first superimposed using Swiss-PdbViewer (26). The sequence alignment generated by the automatic superposition was analyzed manually to identify all homologous positions, maximize the number of aligned residues between as many structures as possible and shift insertions and deletions from conserved secondary structure elements to loops. The alignment revision was guided by direct visual comparison of the structures, including mutual position of compared amino acids and their localization in secondary structure elements.

Sequences of proteins with unknown structures were fitted to the structural alignment generated in the previous step based on secondary structure predictions and alignments returned by the fold recognition methods via the GeneSilico metaserver gateway (27) (for references to original methods see https://genesilico.pl/meta2) and 3D-Jury (23). Because of significant structural divergence of compared proteins in regions outside of the catalytic core, and uncertainty or lack of correct predictions for these regions in proteins of unknown structures, reliable alignment could be generated only for the catalytic core of analyzed proteins. As a result, we obtained a high-quality multiple sequence alignment of a structurally conserved region common to most of RNHL superfamily proteins, comprising two representatives from each group identified during the sequence clustering (Supplementary File S3).

### Structure clustering analysis

The aforementioned 41 representative structures were compared with each other using DALI (28), and the resulting Z-scores were used to build an all-against-all similarity matrix (values <6 were changed to 0). Subsequently, the matrix was clustered using average linkage method and visualized.

### Identification of domains co-occurring with the RNHL domain

For each of the full-length proteins in our data set, we calculated a HMM profile with the HHblits package and used this profile as a query to search a database of Pfam-derived HMM profiles. Based on the nonoverlapping matches with e-values <1e-5, we generated domain composition strings, in which Pfam families were ordered according to their localization in a given protein. The domain compositions strings were compared all-against-all within each RNHL family using the Damerau–Levenshtein distance metric (29). The resulting distance matrices were used for clustering with a Markov Cluster Algorithm, as implemented in (30) (inflation parameter I = 8). The obtained clusters were ranked according to their size and used to identify the most abundant domain architectures in each of the RNHL families. Selected representative proteins were analyzed using the HHpred server (20) and the GeneSilico metaserver (27) (the latter for sequences shorter than the limit of 990 residues). Sequences were divided into independent fragments according to the most confident predictions, and these fragments were resubmitted to prediction servers for identification of individual domain boundaries. Representatives of the RNHL families, which have less common domain architecture, were analyzed using the same procedure.

### Phylogenetic analyses

The phylogenetic reconstruction was conducted using Bayesian phylogenetic inference as implemented in MrBayes (31). The analysis was performed based on the alignment of 304 representative sequences (2 per family) combined with the data on the catalytic residues conservation and the family clustering according to sequence similarity. These additional nonsequence data were obtained and formatted to MrBayes format as follows:

#### Interfamily sequence similarity

For each of the 152 RNHL families defined in this study, a HMM profile was calculated using the HHblits package (all alignments and the corresponding profiles are available for download at ftp://genesilico.pl/iamb/models/RNHL/). Next, all the profiles were compared with each other, and the resulting pairwise *P*-values were used as distance scores for clustering in CLANS. Groups of RNHL families were defined based on the manual investigation of the cluster map obtained with the *P*-value threshold of 1e-2.

#### Conservation of amino acid residues

Manual investigation of the multiple sequence alignments corresponding to the individual RNHL families allowed us to assess the degree of conservation of the following residues: Asp/Glu in the first β-strand, additional Asp/Glu in the first β-strand characteristic for RNases H2, additional Asp/Glu in the first β-strand characteristic for exonucleases, Glu in the first α-helix characteristic for RNases H1, Asp/Glu/His in the fourth β-strand, Asp/Glu in the second α-helix characteristic for exonucleases and Asp/Glu in the fifth β-strand characteristic for resolvases.

Sequence similarity between families was encoded as morphological characters by assigning a unique value to all families belonging to a particular group of families. Conservations of residues were also encoded as morphological characters: ‘1’ was used to indicate that a given position is conserved and ‘0’ that it is not conserved. For each position, additional characters were used to indicate which amino-acid type is conserved: for example, conservation of Asp in the fifth β-strand would be encoded with three separate characters, 1-1-0: first character (1) indicates that the position is conserved (regardless of the amino-acid type at this position), second character (1) indicates that Asp is the most frequent amino-acid at this position (>50% of occurrences in the multiple sequence alignment column) and the third character (0) indicates that Glu, another residue that tends to be present at this position, is infrequently observed (<50% of occurrences). For a detailed description of morphological characters, see Figure 4 and Supplementary File S4.

To balance the relative weight of the sequence and nonsequence data used for the phylogenetic reconstruction, the amount of morphological characters columns was multiplied: a single column defining interfamily sequence similarity was multiplied 20 times, whereas 20 columns describing residue conservation were multiplied three times. This arbitrary assignment of weights to different types of data was based on our experience with similar analyses in the past (32) and aimed at ensuring comparable impact of the sequence and nonsequence data (interfamily distances and catalytic core conservation) on the results of the phylogenetic analysis. All MrBayes simulations were run for 10 000 000 generations. Average standard deviation of split frequencies <0.05 was considered as an indicator of simulation convergence. Trees were generated after discarding the first 25% of samples. To check whether sequence and nonsequence data bear a consistent evolutionary signal, we performed two additional MrBayes analyses, first based on the multiple sequence alignment alone, and second based on the interfamily similarity and conservation data sets. Deep branches in the two resulting trees were not well supported (see Supplementary Files S6 and S7); however, both comprise similar sequence groups. The tree calculated based on the combined sequence and nonsequence data also formed similar groups, but yielded considerably better support of the deep branches (Supplementary File S4 contains the MrBayes input file and Supplementary File S5 contains the phylogenetic tree shown on Figure 6). As additional validation of our approach, we tested the following combinations of weights and sequence/nonsequence data sets: (i) multiple sequence alignment and the interfamily sequence similarity score, (ii) multiple sequence alignment and the conservation score and (iii) multiple sequence alignment and both scores, but with reversed weights (i.e. interfamily sequence similarity character was multiplied 55 times, whereas residue conservation characters were not multiplied). We found that all analyses performed for the above data sets resulted in trees yielding similar groups (Supplementary Files S8–S10). We therefore conclude that our approach is robust and that the definition of clades presented on Figure 6 is not biased by the selection of data sets and weighting.

## RESULTS AND DISCUSSION

### Identification of known and new RNHL superfamily members

To collect all known RNase H-like families, including those without structurally characterized representatives, and to identify other potentially homologous families, we conducted extensive sequence database searches. We used the representative sequences as queries to search profile-HMMs corresponding to multiple sequence alignments of protein families obtained from the COG, KOG (18) and Pfam (19) databases, using the HHpred server (20). Based on the results of the HMM-HMM comparison, we generated a preliminary list of new candidate RNase H-like families. The preliminary candidates were initially validated by reciprocal HHpred searches against the database comprising all the COG, KOG and Pfam profile-HMMs. Further, they were used in transitive Meta-BASIC (22) searches for new superfamily members among all Pfam, COG and KOG families. This approach was successfully used in our previous studies on highly diverged NTase fold (33) and PD-(D/E)XK phospohodiesterase (34) superfamilies. To consider the observed high sequence divergence in RNHL superfamily reflected in scores lower than the default Meta-BASIC threshold, we also analyzed hits with scores lower than the threshold. This resulted in the identification of 13 Pfam and COG families (PF14239, PF04312, COG2433, PF11215, PF04250, COG2410, COG4328, COG4923, PF06485, COG5558, PF01385, COG0675, PF05380) that represent novel potential RNHL domains, which escaped detection with other advanced homology search methods. If a region of sequence that initially seemed to be similar to known RNase H-like members displayed significant similarity to another family, known to be unrelated to RNase H (e.g. based on the knowledge of the experimentally determined structure), then a given family was regarded as a false positive and removed from the list of RNHL superfamily candidates. Each candidate family, for which the relationship to known RNase H-like families was confirmed by reciprocal searches, was also analyzed by fold-recognition method via the GeneSilico metaserver (27) and 3D-Jury (23). This step served to evaluate the compatibility of the query sequence with the known RNase H-like structures, and to detect cases, where other unrelated structures could be found as better matches. Thereby, we eliminated proteins with evident similarity to other members of the RNase H-like fold, but corresponding to different superfamilies according to the SCOP database, e.g. actin-like ATPases, creatinase/prolidase N-terminal domain, ribosomal proteins L18 and S11 and their homologs, nitrogenase accessory factor-like proteins, homologs of domain II from the DNA repair protein MutS and the methylated DNA–protein cysteine methyltransferase domains. This step also allowed us to determine if potential active site residues were present in positions corresponding to positions of known catalytic residues in the RNHL superfamily. The validated COG/KOG clusters and Pfam domains have been used in further steps of the analysis. To our knowledge, many of these proteins (including those that belong to the 13 newly identified Pfam and COG families, as well as those not yet assigned to any Pfam or COG) have not yet been assigned to the RNHL superfamily.

To identify a complete set of the RNHL superfamily members (beyond proteins included in the COG, KOG and Pfam alignments), we conducted clustering of the sequences retrieved from those databases, selected an extended set of representatives and used the sequences of their RNase H-like domains (with other domains removed) as queries in PSI-BLAST searches of the nr database, carried out until convergence. We retrieved all sequences reported with e-value < 1e-3 and removed obvious duplicates. We have also conducted extensive analysis of the available literature to find all potentially homologous proteins, including highly divergent proteins that were not identified by the methods described above. In the final data set, we have included transposases and their inactive derivatives, 3′–5′ exonuclease domains of numerous enzymes including DNA polymerases as well as RNases, some of which, though not all, have been included in the RNase H clan in the Pfam database.

### Newly identified RNHL families

Transposases constitute the most abundant group of RNHL families (35). We identified several families of known and putative transposases as RNHL superfamily members: OrfB_IS605 (PF01385 and corresponding COG0675), DDE_Tnp_Tn3 (PF01526, COG4644), DUF1092 (PF06485) and COG5558. Interestingly, COG5558 proteins are present solely in Archaea, DUF1092 consists of hypothetical proteins of unknown function all from photosynthetic organisms including plants and cyanobacteria and according to the phylogenetic analysis performed in this study are related to transposases and integrases. OrfB_IS605 insertion sequences are found in prokaryotes and their phages. DDE_Tnp_Tn3 transposases were most likely horizontally transferred from soil bacteria both to plants (*Ricinus communis* GI: 255594834, 255599154 and *Populus balsamifera*, GI: 222834205) and fungi (*Sordaria macrospora*, GI: 336241630).

Members of the PF05380 family (Peptidase_A17 in Pfam) are present in Bel/Pao LTR retrotransposons. This protein family exhibits a patchy phylogenetic distribution in eukaryotes, which is a feature characteristic for mobile genetic elements. A typical member of PF05380 retains catalytic residues typical for RNase H1 and most likely functions as a nuclease of the reverse transcriptase protein, as it often follows the RVT_1 reverse transcriptase domain within hypothetical Bel/Pao *pol* polyproteins (encompassing DUF1758 aspartic protease, RVT_1 reverse transcriptase domain, peptidase_A17 RNase H domain and rve integrase). The former classification to the A17 protein family of Bel/Pao peptidases in MEROPS database (36) thus seems to be invalid according to our predictions.

We found that two other protein families: RRXRR (PF14239) and DUF429 (PF04250 and COG2410, COG4328, COG4923) that encompass uncharacterized and poorly annotated proteins, should be also assigned as members to the RNHL superfamily. The genomic context of DUF429 is conserved in closely related taxa, which suggests that these proteins are unlikely to be mobile. These families display conservation of potential active site residues within the RNHL domain (in the first and fourth strand), suggesting that these proteins are likely to be active as nucleases. The RNHL domain of PF14239 proteins contains a RRXRR motif in the highly positively charged region directly preceding the first core α-helix. Genomic context of proteins belonging to this family is highly variable, not even conserved between different strains of one species (Anabaena, Polaromonas). Interestingly, two HGTs could be identified from bacteria to Archaea (*Methanohalobium evestigatum*, GI: 298674346, 298674707, 298675090, 298675173, 298675374) and from bacteria to plants (*Taxus wallichiana*, GI: 16611907).

Two other domains of unknown functions, DUF460 (PF04312/COG2433) and DUF3010 (PF11215), not assigned to RNase H clan in Pfam, but predicted herein to belong to the RNHL superfamily, show similarity to HJRs. DUF460 is present solely in Archaea, it has conserved classic RNHL active site residues (the aspartates in first and fourth strand, and in C-terminal helix) and it is also related to the Toxin expression protein (Tex) family. DUF3010 comprises single-domain bacterial proteins (mostly from Gammaproteobacteria). It exhibits conserved active site residues similar to the active site of exonucleases.

Transposase families that have been identified previously, but not assigned explicitly to the RNase H clan, include DUF1258 (PF06869), Transposase_21 (PF02992), DUF1280 (PF06918), Tnp_P_element family (PF12017), DUF659 (PF04937) and DUF4371 (PF14291). DUF1258 (PF06869) encompasses proteins from animals, mostly nematodes. This family corresponds to the Mirage group of putative transposases described previously (35). DUF1258 family members are relatively close homologs of Transposase_21 (PF02992) proteins, which clustered together with CACTA (En/Spm) transposases. Transposase_21 proteins are present mostly in plants, with members horizontally transferred to *Bifidobacterium breve* (GI: 291457831). Both DUF1258 and Transposase_21 have conserved active site residues similar to RNase H-like proteins, which suggests that they are functional transposases. DUF1280 (PF06918) corresponds to putative transposases of Chapaev transposons present in animals (35).

DUF659 (PF04937) and DUF4371 (PF14291) include transposases of hAT transposons. DUF659 is found mostly in Viridiplantae and it has conserved RNHL active site residues, thus most likely is a functional transposase. DUF4371 proteins occur in transposable elements (both in DNA transposons and retroelements) and their phylogenetic distribution in eukaryotes is patchy (e.g. they are absent in plants). DUF4371 proteins are composed of an N-terminal ZnF_TTF domain (acronym for ‘zinc finger in transposases and transcription factors’) followed by an RNHL domain. DUF4371 family members usually have additional domains including other zinc fingers (THAP, zf-FCS) located at the N-terminus and a hAT family C-terminal dimerization region (Dimer_Tnp_hAT). In Pfam database the Tnp_P_element family (PF12017) comprises sequences from insects, yet we find its distant homologs also within Amebozoa (*Polysphondylium pallidum*, GI: 281203306) and Chromalveolata (*Aureococcus anophagefferens*, GI: 323449162).

Terminases are phage proteins involved in DNA packaging and phage assembly [review: (37)]. We found that several nuclease domains of phage terminases, including Terminase_1 (PF03354), Terminase_3 (PF04466), Terminase_6 (PF03237), DNA_pack_C (PF02499) and the Terminase_GpA family (PF05876), are assigned to the P-loop NTPase clan in the Pfam database. All these Pfam families contain both an RNHL domain and a P-loop NTPase domain located N-terminally to the RNHL domain, with the exception of DNA_pack_C (PF02499), which comprises only the RNHL domain and usually has DNA_pack_N (PF02500) domain located N-terminally. GpA terminases are bacteriophage tail assembly proteins, which in most cases harbor no additional domains.

#### Clustering analysis of the RNHL superfamily

Clustering of RNHL domain sequences was performed based on their pairwise BLAST similarity scores using CLANS (17). The clustering resulted in identification of 152 clusters comprising 60 923 sequences (1000 sequences were not assigned to any cluster) (Figure 1, Supplementary File S2, Supplementary Table S1). An overview of these families is given below.

#### RNases H

RNases H type 1 (largely included in PF00075, PF13456, COG0328, COG3341 and KOG3752) grouped into eight clusters, strongly connected to each other. Clusters E.1, E.3, E.4 and E.5 embrace eukaryotic, bacterial and viral proteins, including those from gammaretroviruses and endogenous viruses similar to gammaretroviruses, epsilonretroviruses and spumaviruses. RNHL domain of Ty3/Gypsy retrotransposons grouped within clusters E.4 and E.5. Sequences of the RNase H domain of non-LTR retrotransposons formed a cluster E.7. RNase H sequences from lentiviruses grouped into one cluster E.8. RNase H domain of the Betaretroviruses clustered in E.11 with mammalian Alpharetroviruses, avian Alpharetroviruses and Deltaretroviruses. RNase H domain of Ty1/Copia retrotransposons formed a large cluster E.2 that is connected but clearly separated from the other seven clusters. The more diverged sequences of RNase H domain from the C-terminus of hepatitis B-type viruses P proteins (PF00336) grouped in distant cluster E.15. Most of the putative nucleases of the reverse transciptase from Bel/Pao-like elements (PF05380) formed a cluster E.6.

RNase H type 2 enzymes (RNH2 and RNH3) (PF01351) grouped together into one cluster IV.1 without strong sequence similarity to the above-mentioned RNases H1. Within that cluster we distinguished three subclusters: prokaryotic RNases H2 (COG0164), RNases H2 from Eukaryota (KOG2299) together with RNases H2 from Archaea (COG0164) and RNases H3 (COG1039).

#### Integrases/transposases

The integrase core domain (RVE, PF00665, PF13333, PF13683) sequences are present in 14 clusters (A.1, 2, 5, 7, 11, 12, 14, 15, 17, 18, 19, 21, 25 and 27) connected with each other to form a supercluster in the central part of the clustering diagram. Sequences of integrase domain of Ty3/Gypsy-related retrotransposons grouped within clusters A.2 and A.12, while Ty1/Copia retrotransposons integrase can be found in cluster A.5 that formed on the edge of the central supercluster. According to the phylogenetic analysis (see below) these clusters belong to a monophyletic group that also contains transposases (contained in clusters A.3, 4, 6, 8, 9, 10, 13, 16, 17, 20, 22, 24 and 28). Some of these clusters contain both, integrases and transposases, further confirming strong similarity between these groups. This finding is in agreement with previous studies that revealed similarity between retroelement integrases and DDE transposases from Mariner transposons (38).

DUF4371 (PF14291, B.2) and DUF659 (PF04937, B.3) and P element transposase (Tnp_P_element, PF12017, B.4) show similarity to hAT Activator transposase. Transposase_21 (PF02992) and DUF1258 (PF06869) families clustered together (III.1). Tn3 transposaes (PF01526, COG4644) formed cluster III.3.

OrfB_IS605 family members (PF01385, COG0675) show no strong similarity to any other family in the clustering of a phylogenetic analysis. Clusters of different transposases are listed in Supplementary Table S1.

#### Other endonucleases

The DDE endonuclease (PF03184) cluster A.4 can be divided into two subgroups, one comprising mainly predicted fungal proteins of unknown function, and the other comprising Tigger transposable element derived proteins, Jerky protein homologs as well as the Centromere Protein B domain (KOG3105). The latter appears to have lost the metal binding residues, thus is unlikely to have endonuclease activity (39).

HJRs and related proteins formed six well-defined clusters. RuvC endodeoxyribonucleases (PF02075, COG0817) and the mitochondrial resolvase Ydc2 family (PF09159) formed clusters D.13 and D.2, respectively. The Poxvirus A22 protein (PF04848), a HJR of the dsDNA viruses (mostly Poxviridae), and PF07066 family that comprises several *Lactococcu*s phage middle-3 (M3) proteins, formed small clusters D.24 and D.25, respectively, but some of the individual sequences remained spread between clusters. The YqgF/RuvX family of putative HJRs (PF03652, COG0816) split into two well defined and strongly connected clusters (D.1 and D.14). Both clusters are connected to cluster D.7 that represents Transcriptional accessory or Toxin expression protein (Tex) (COG2183, KOG1857), which in turn is connected to cluster D.9 of Transcription elongation factor SPT6 (COG2183, KOG1856). Sequences of DUF460 (PF04312, COG2433, cluster D.18) were also clustered close to the above-mentioned groups. Sequences of COG3513 (predicted CRISPR-associated nucleases that contain McrA/HNH-nuclease and RuvC-like nuclease domains), comprising Cns1 and Cas5e families of CRISPR-associated proteins form tight separate cluster D.16 and also showed similarity to the RuvC and RuvX HJRs. Sequences from the PF11215 (DUF3010, cluster D.19) family, which grouped together gammaproteobacterial proteins of unknown function also showed similarity to HJRs and Tex proteins.

Piwi/Argonaute domain sequences (PF02171) formed a large cluster with a central part comprising eukaryotic proteins (KOG1041—Translation initiation factor 2C (eIF-2C) and related proteins, KOG1042—Germ line stem cell division protein Hiwi/Piwi), surrounded by smaller subgroups of bacterial and archaeal sequences from COG1431.

The endonuclease V sequences (PF04493, COG1515) formed I.2 cluster localized on the edge of the clustering map, but with more permissive threshold values it showed connections to the nuclease subunit of the excinuclease complex—UvrC protein (PF08459, COG0322, cluster I.1). Sequences annotated either as the Endonuclease V homologs (COG1628) or proteins of unknown function DUF99 (PF01949) formed cluster V.1.

Families annotated as the nuclease domain of phage terminases (PF05876, COG5525, PF03237, PF04466, PF03354, COG4626, COG5362, COG5323, COG4373 and PF02499) formed seven separate clusters (III.4, III.2, D.4, D.5, D.8, D.17 and D.23).

Proteins from the DUF458 (PF04308, COG1978) family represented by the *B. subtilis* YkuK protein, already predicted to be members of the RNHL superfamily (40), formed a disconnected cluster E.9, and showed a few connections to different groups when analyzed with more permissive threshold values.

A group of proteins assigned to the NurA domain family (PF09376) formed four clusters (C.18, C.21, C.22 and C.27).

Cluster V.2 comprising the RNase H-like domain of the pre-mRNA processing splicing factor 8 (prp8) (PF12134, COG5178, KOG1795) was localized on the edge of the clustering diagram and does not show significant sequence similarity to any of the other families. RAG1 proteins (products of the recombination activating gene 1) (PF12940, cluster II.2), involved in V-D-J recombination, also separated at the early stage of the clustering process.

#### 3′–5′ exonucleases

A large cluster F.3 was formed by the PF03104 (DNA polymerase family B, exonuclease domain) with eukaryotic DNA polymerases alpha, delta, epsilon and zeta (KOG0970, KOG0969, KOG1798, KOG0968) and prokaryotic DNA polymerase II (COG0417). This cluster is tightly connected to the cluster F.12 of predicted 3′–5′ exonucleases related to the exonuclease domain of PolB (PF10108, COG3298). Strongly connected to both of these clusters were other three clusters with members of PF13482: the first harbored predicted exonucleases from COG3359 (F.10), the second grouped predicted nuclease (RecB family) from COG2251 (F.13) and the third grouped sequences of PF13482 (F.23) from various phages. PF13482 members were also found in a separate cluster comprising RecQ ATP-dependent DNA helicases (DEAD/DEAH-box like helicase) and T7 DNA polymerases (F.14). DNA polymerases type B from plant and fungal mitochondria and viruses (PF03175) split into four small clusters based on the organism of origin (F.19, F.20, F.22, F.29).

The PF00929 family, which included a variety of exonuclease proteins, formed one large cluster surrounded by five strongly connected clusters of variable size. The largest cluster F.1 comprised a variety of exonucleases, including RNase T, epsilon and alpha subunits of the DNA polymerase III, probable ATP-dependent helicase DinG homolog, 3′ repair exonuclease 2 (TREX2) (COG0847, COG2176, COG1199, KOG4793) and some sequences of PF13482. We also distinguished a cluster F.8 that comprised KOG0542 and COG5018, proteins like Enhanced RNA-interference protein 1 (Eri1), 3′ histone mRNA exonuclease 1 (THEX1), Cell death Related Nuclease 4 (CRN4), Inhibitor of the KinA pathway to sporulation (sporulation inhibitor KapD) and the Prion protein interacting protein 1 (PRNPIP). There were also two small strongly connected clusters of PF00929, F.15 with Exodeoxyribonuclease I (COG2925) and F.21 with Exodeoxyribonuclease VIII enzymes. Oligoribonucleases (KOG3242, COG1949) formed a separate cluster F.7. The last large separate cluster of PF00929 (F.5) comprised RNA exonucleases 1, 3 and 4 (REXO1, REXO3, REXO4), interferon-simulated gene product of 20 kDa protein (ISG20) (KOG2249), Exonuclease NEF-sp (KOG2248, COG0847) and the deadenylating RNase PAN2 (PAB-dependent poly(A) RNase, subunit PAN2) (KOG1275).

The deadenylating enzymes (CAF1 family RNase, PF04857) grouped into two clusters: F.9 comprising POP2 and CAF1 nucleases (KOG0304, COG5228) and F.11 with the poly(A)-specific exoribonuclease Poly(A)-specific RNase (PARN) (KOG1990) and the TOE1 protein (target of EGR1). Members of DNA_pol_A_exo1 (PF01612) (one of the Pfam families representing 3′–5′ exonucleases) formed several clusters. DNA polymerase I 3′-5′ exonuclease domain (COG0749) formed one large cluster F.2. Next to it there was a second group representing PF01612, which we divided into four subgroups: F.6 comprised exonucleases from KOG2207 and KOG4373, including the Warner syndrome ATP-dependent helicase (WRN); F.4 comprised RNase D (COG0349) and exosome complex exonuclease Rrp6 (KOG2206); F.16 comprised uncharacterized exonucleases from KOG2405; and F.25 comprised sequences of uncharacterized exonucleases from *Caenorhabditis*.

Cluster VI.3 of maelstrom proteins (PF13017) involved in germ line piRNA pathway, at more permissive threshold values showed some connections to 3′–5′ exonucleases, mainly DNA polymerase III. An RNase H-like domain from the Poxvirus F12L protein (Pox_F12L) (PF03337, cluster F.28), assigned to DNA polymerase B-like clan in Pfam, showed remote connections to several exonucleases. Arenavirus nucleocapsid proteins (PF00843, cluster F.24) also showed similarity to exonucleases. The phylogenetic analysis (see below) confirmed that these clusters are included in (or are related to) the exonuclease clade F.

### Structural features of known members of the RNHL superfamily

**Figure 2.**
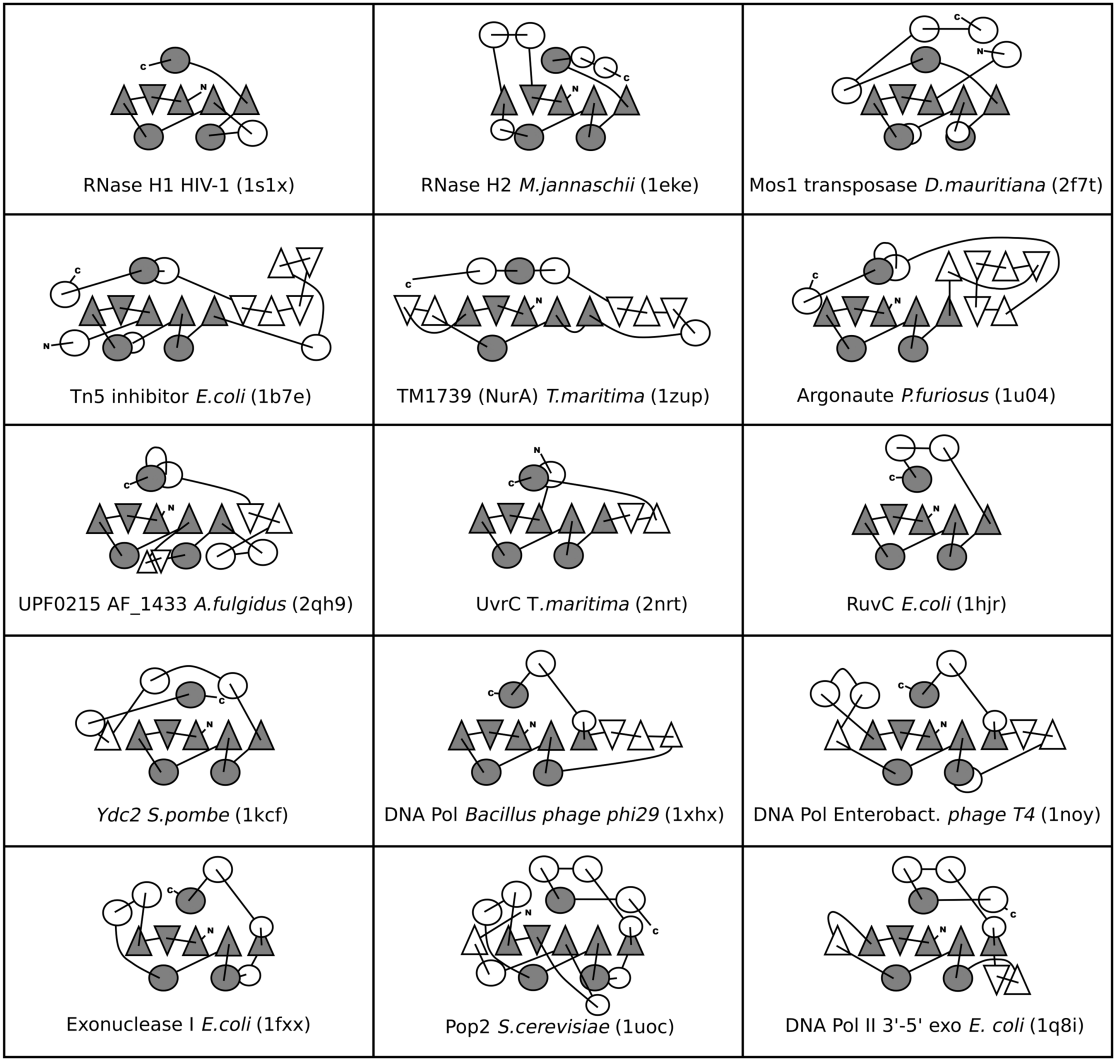
Topological diagrams of representative RNHL superfamily structures. α-helices are shown as circles, β-strands are shown as triangles. Orientation of the triangles shows the orientation of the β-strands, with vertex of the triangle pointing up for the β-strand pointing toward the reader, and vertex of the triangle pointing down for opposite orientation of the β-strand. Universally conserved elements are shown in gray, variable elements are in white.

To identify all members of the RNHL superfamily, extended searches of the structure and sequence databases were conducted (as described in ‘Materials and Methods’ section). Comparison of the selected structural representatives required a correct superposition of the structures. Due to high divergence of the structures, the superposition had to be conducted manually, based on the knowledge of relative positions of corresponding catalytic amino acids. To compare topologies of the representative domains, we prepared topological diagrams representing the structures (selected examples presented in Figure 2), showing relative positions of the secondary structure elements, their direction and the way they are connected.

Comparison of RNHL domains revealed the presence of a common core, comprising a mixed β-sheet of five β-strands ordered 32 145, with β2 antiparallel to the other β-strands, and three α-helices. Two of these structurally conserved helices (α1 and α2) are present between the β-strands in the sequence on one side of the β-sheet, while the third structurally conserved α-helix (α3) is located in the C-terminus on an opposite side of the β-sheet. The C-terminal α-helix of the catalytic core can be found either immediately after the β5 strand of the core or separated from it by an insertion of variable size. The length of the strands differs vastly, both within and between different members of the RNHL superfamily. Strands β1, β2 and β3 are typically longer (8–10 residues) than strands β4 and β5 (3–6 residues). The examples of the exceptions are the structures 1zup (TM1739) and 3e9l (RNHL domain of Prp8), where the first β-strands (three and two, respectively) are almost 20 residues long. In 1zup the extension forms an additional β sheet with two small strands inserted after the common strand β3, while in 3e9l the first two β-strands form a β-finger that protrudes from the protein. The common α-helices maintain their approximate position with respect to other secondary structure elements; however, they vary greatly in size and in the angle at which they are positioned relative to other helices and to the central β-sheet; they often cannot be simultaneously superimposed, if strongly divergent members are compared with each other.

In many members of the superfamily, additional secondary structure elements (most often at least one α-helix) are present N-terminally to the core, but they are not conserved throughout the superfamily and can be found in different positions and orientations with respect to the core. The C-terminal part of the RNHL superfamily members shows even higher variability than the N-terminus. Additional α-helices can be present there; they may assume positions on both sides of the central β-sheet, but are more common on the side corresponding to the position of the C-terminal α-helix of the core (α3). Such expansion of the core is present in RNases H2 and H3 (PDB codes 1eke, 2d0a), HJRs (1hjr, 1kcf, 1vhx) and numerous 3′–5′ exonucleases, especially deadenylating RNases (1uoc and 2a1r). In many members the central β-sheet is extended by additional β-strands that can be found next to β3 or β5, at both edges of the common β sheet. In the Piwi/Argonaute proteins (1u04, 1w9h), Tn5 transposase inhibitor (1b7e) or 3′–5′ exonuclease domain of *E. coli* DNA polymerase II (1q8i), not only an extension of the core β-sheet is present, but also an additional β-sheet parallel to the core β-sheet.

Topological diagrams illustrate the presence of structural elements characteristic for particular groups of more closely related proteins. Insertion of α-helices between the strands β2 and β3 is characteristic for RNases H2 and H3. In other proteins, insertions between the first three β-strands or between the first α1 and β4 are relatively rare. Insertion of additional secondary structure elements, usually α-helices, between β3 and α1 is common in exonucleases. This region has been shown to carry residues important for binding of the substrate 3′-terminus, indicating its importance for exonuclease activity (41,42).

An interesting feature observed in some of the RNHL superfamily members is an inversion of the C-terminal α-helix, which preserves the axis of the helix, but reverses the direction of the polypeptide. In RNases H, integrases, transposases as well as in Ago/Piwi, UvrC, EndoV, Prp8 and also in NurA, the direction of the polypeptide in the C-terminal helix is the same as in β1, so the C-α goes along the β1 from its N to its C terminus. In DNA polymerases, deadenylases and many other exonucleases, including RNaseT, ExoI, Eri1, but also in some endonucleases including terminases and HJRs, the C-terminal helix runs in the opposite direction. The structure of *B. halodurans* RNase H1 (2g8h) lacks a regular C-terminal α-helix, which is replaced a by a loop without secondary structure, but the catalytic Asp residue is still present and positioned similarly to other members. On the contrary, in the reverse transcriptase connection domain, the helix, the loop or the catalytic residue is not present. In this case, β5 is linked directly to the subsequent domain. Another interesting feature that distinguishes 3′–5′ exonucleases from other RNHL superfamily members is the direct transition of the fifth β-strand into an α-helix.

### Sequence alignment and conservation of the representative proteins

To make the comparative analysis of the entire RNHL superfamily feasible with standard tools for phylogenetic analysis, we selected a set of representative sequences. Our aim was to maximize the coverage of sequence, structure and functional diversity in this superfamily. Based on the multiple sequence alignment generated for each of the identified groups described above, we selected two representative sequences from each major cluster. In the selection of the first representative we gave preference to sequences with known structures and from the SwissProt section of the UniProt Knowledgebase, which includes human-curated functional annotations. For the second representative, we selected sequences that were relatively dissimilar to the first representative, to maximize the content of information about sequence divergence within the family. As a result, we gathered 304 representative sequences of the RNase H and RNase H-like domains extracted from 152 clusters and subjected them to further analyses.

**Figure 3.**
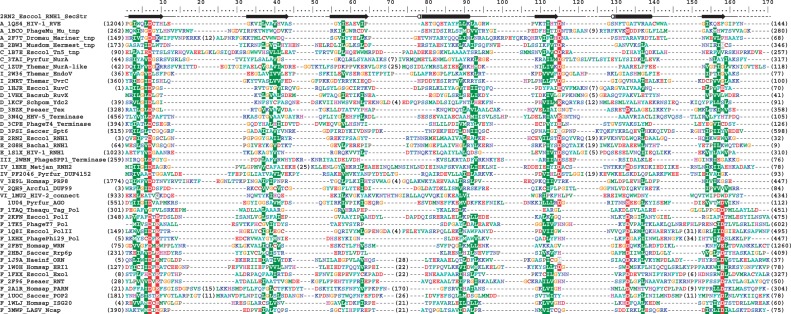
Structure-based multiple sequence alignment of the core of the RNase H-like domain of the 41 structural representatives. Sequences are denoted by a letter indicating their clade (described in this work), PDB code, six-letter abbreviation for genus and species and a common name, abbreviated in most cases. Residues are colored by physicochemical properties of their side chains, and background-colored positions are those showing at least 40% identity/similarity. The variable termini and insertions are not shown; the number of omitted residues is indicated in parentheses. Secondary structure elements determined for selected representative are shown above the alignment, where α-helices are represented by tubes, β-strands by arrows and loops by continuous lines.

The RNase H fold is one of the evolutionarily oldest protein folds (9), and in the course of divergent evolution sequences of its members accumulated numerous substitutions, insertions, deletions and underwent fusions with various domains. Due to this divergence, sequence similarity between different families of RNHL proteins is low, often undetectable with standard methods. The length of the RNase H-like domain in different proteins often varies significantly owing to the presence of numerous insertions in the catalytic core. Expectedly, a multiple sequence alignment of the representatives that we generated in a fully automated manner was evidently incorrect, as it failed to match many structurally and functionally corresponding residues and motifs (data not shown). Therefore, we generated the alignment by hand, based on the analysis of known structures, secondary structure predictions and pairwise alignments between proteins of unknown and known structure, returned by the fold recognition methods. First, a structure-based sequence alignment of the 41 representative proteins with known structures was generated based on spatial superposition of these structures (Figure 3) (see ‘Materials and Methods’ section for details). Subsequently, we added individual sequences of proteins with unknown structures, based on structural predictions, with additional constraints on superposition of functionally equivalent residues (e.g. those of the active site). As a result, we obtained a high-quality sequence alignment of the catalytic core of the RNHL domain from all of the identified families (Supplementary File S3).

Because of strong sequence divergence of various members of the RNHL superfamily, there are no evidently conserved sequence motifs or patterns common to all the RNase H-like families, apart from the position of catalytic amino acid residues. However, even these residues are not absolutely conserved. The overall conservation of the physicochemical properties of residues in the core (i.e. hydrophobicity) is a general feature of globular proteins. Nonetheless, the alignment hints at sequence similarities between certain families of RNase H-like proteins that may be more closely related to each other than to other members.

**Figure 4.**
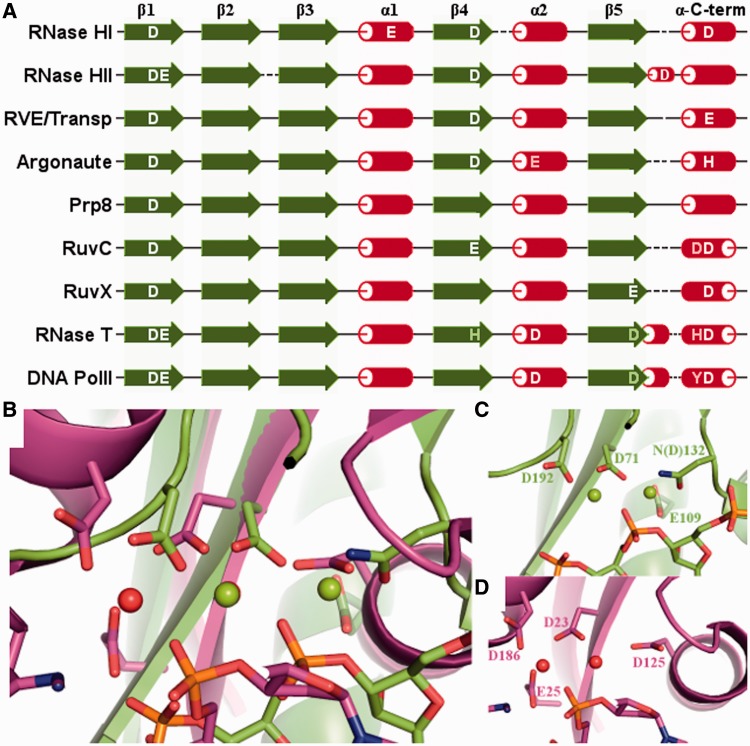
Configuration of the active site. (**A**) Known and predicted catalytic residues in exemplary RNHL families with respect to the secondary structure of the catalytic core. Catalytic residues are shown in white. Conserved residues important for the activity but not directly involved in catalysis are shown in gray. (**B**) Superposition of the active sites of RNase H1 (D132N mutant) from *B. halodurans* (PDB code: 1zbi; shown in green) and RNase T from *E. coli* (PDB code: 3nh1; shown in magenta), with metal ions and substrate nucleic acids bound. (**C**) Active site of RNase H1 from *B. halodurans* (in the same orientation as in panel B). (**D**) Active site of RNase T from *E. coli* (in the same orientation as in panel B).

The position of some of the catalytic residues is, expectedly, highly conserved among members of the RNHL superfamily. However, there are numerous variants of the active site, characteristic for specific subgroups, differing by the position and identity of additional catalytic residues. Due to higher flexibility of the α-helices in the RNHL fold compared with β-strands, the position of the catalytic amino acid residues located in α-helices is slightly more variable. They seem to be less critical for the enzymatic activity than the ones localized in the β-strands, and the main feature determining their functionality is a proper distance from the other catalytic residues. In some cases, the active site is rearranged or incomplete, lacking one or more of the catalytic residues, which often makes these enzymes inactive. Comparison of the structures in the complexes with the substrates and metal ions shows that the position of catalytic residues and metal ions is relatively well conserved within endonucleases, and even more conserved within exonucleases. When these two groups are compared with each other, we observe a shift in the position of the active site and the metal ions with respect to the core of the RNHL fold (Figure 4).

The position of the first aspartic acid residue, located in the central part of strand β1, is conserved in all of the RNHL enzymes. In RNase H2, an additional E residue is present right next to the first catalytic D. An acidic residue in this position is also present in some of the transposases and Prp8, and E is sometimes replaced by D. The RNase H-like domain of Prp8 is an example of an incomplete active site, in which only the carboxylate chains in strand β1 (DD corresponding to DE in RNase H2), are retained in the putative active site. It has been hypothesized that this domain may form a composite nuclease active site together with the functional groups from the bound RNA substrate (8). Additional glutamic acid in strand β1, separated from the first catalytic residue by one other residue, is characteristic for 3′-5′ exonucleases. Additional glutamic acid in helix α1 of the core is characteristic for RNase H1, but also present in Endonuclease V, NurA and YkuK. Although NurA has been shown to have both the endo- and exonuclease activity (43), the architecture of its active site as well as the direction of the C-terminal α-helix are characteristic for endonucleases.

There are two alternative variants of the position of the second canonical catalytic residue in the active site. In 3′–5′ exonucleases, this catalytic residue is localized in the N-terminal part of helix α2, while in the other enzymes from this superfamily is at the C-end of strand β4. All three residues, D, E and H, are relatively common in the catalytic position in strand β4, while in helix α2, only D or E are observed. Many exonucleases have a conserved residue, usually H or Y, in the position in β4, in addition to the catalytic residue in helix α2. However, in these cases, the side chain of H or Y often faces away from the active site and its role was predicted to be structural rather than directly involved in catalysis (44). In the case of Argonaute/Piwi domains, the aspartic acid is present in strand β4 and has been confirmed to be critical for catalysis (45). A glutamic acid residue in helix α2 is also present in Argonaute/Piwi and has been claimed to be the third catalytic residue (5,6). However, mutagenesis studies did not confirm that hypothesis, as the mutant proteins retained the ‘slicer’ nuclease activity (46), and confirmed that the third catalytic residue is located in the canonical position of the C-terminal α-helix (46,47). As predicted by Aravind *et al.* (48) and subsequently confirmed by crystallographic studies, RuvX (YqgF) (PDB code: 1vhx) (49) and Tex (PDB code: 3bzk) (50) are the examples of the active site rearrangement, where the conserved acidic residue is relocated from the typical position in β4 to a nonhomologous position in β5. In Tex, the migrated carboxylate group maintains its position despite its different location in the fold, nevertheless this protein lacks the third carboxylate, which usually comes from the C-terminal α-helix. Conversely, YqgF retains all three conserved carboxylates; however, the aspartic acid residue, which migrated to β5, is bent away and unlikely to form a functional active site. Thus far, the nuclease activity has neither been corroborated for Tex nor for YqgF (50,51). In the C-terminal part of β5 of exonucleases, a conserved D is found, sometimes replaced by E. It has been shown that mutation of that residue causes a significant reduction in exonuclease activity (52,53). However, the role of that residue appears to be more in maintaining the local structure rather than directly in catalysis (52,54).

**Figure 5.**
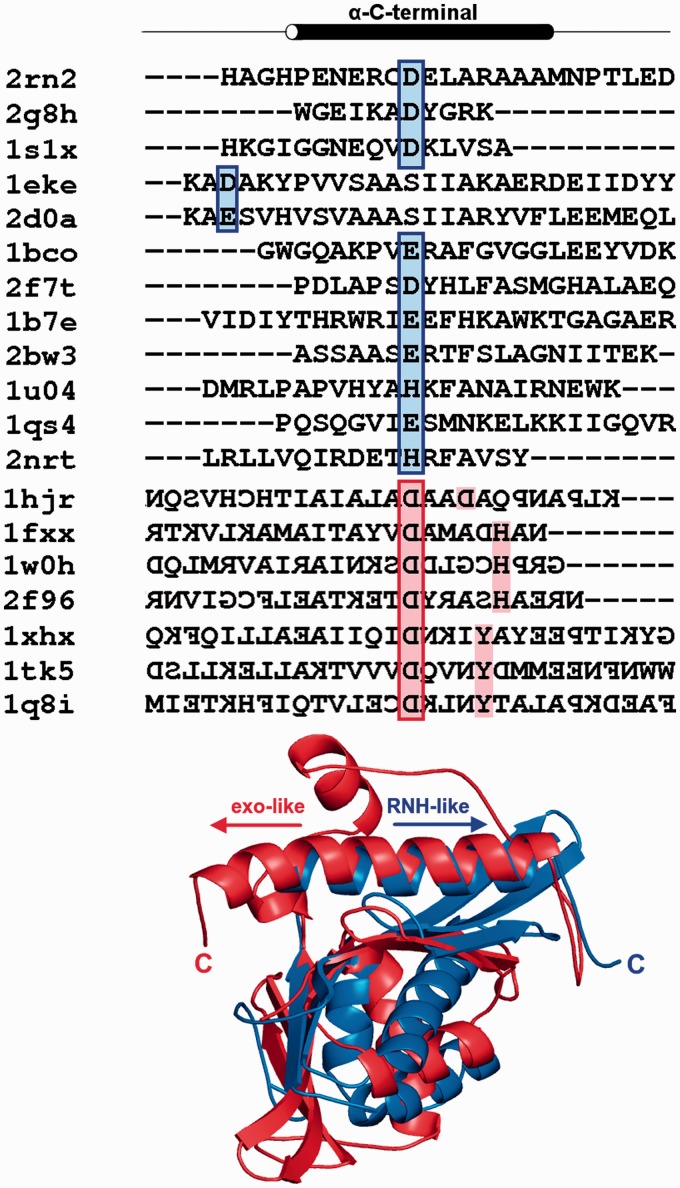
Sequence alignment of the exemplary C-terminal α-helices of the catalytic core and comparison of its two alternative orientations. Sequences are denoted by the PDB code. Position colored in red and blue indicates position of the last catalytic amino acid. Direction of the α-helix from N- to C-terminus is indicated by arrows.

The third canonical catalytic residue of the RNHL superfamily is located in the C-terminal α-helix of the catalytic core. Regardless of the inversion of the direction of the polypeptide in the C-terminal α-helix, the position of this catalytic residue is conserved with respect to other catalytic residues. The only well characterized exceptions are RNase H2 and H3, in which this catalytic residue is shifted to the short 3/10-helix between the β5 strand and the C-terminal α-helix. In the enzymes with the C-terminal α-helix oriented as in RNase H, residues D, E or H can be found in the position of the third catalytic residue. In enzymes with the exonuclease-like orientation of the C-terminal α-helix, D is typically found in a spatially corresponding position. Interestingly, the enzymes of the latter type often have an additional conserved residue in that α-helix. HJRs have an additional D, located three residues N-terminally to the canonical D, while exonucleases have either an additional Y located four residues N-terminally, or an H, located five residues N-terminally. These additional conserved residues presumably play a role in activating a water molecule, which then attacks the phosphorus atom in the target phosphodiester bond (55). The sequence alignment of the C-terminal α-helix of exemplary proteins is shown in Figure 5. It could not be prepared for the proteins with unknown structures, as it is common for the C-terminal helix to be separated from the core by a different number of additional helices and its position often cannot be predicted accurately. Also, due to inverted orientation of the helix in different proteins, the conservation of the last catalytic amino acid could not be shown using a standard alignment.

### Phylogenetic analysis

To reconstruct the evolutionary events that gave rise to the 152 RNHL families defined by the clustering procedure and to reveal relationships between them, we carried out a phylogenetic reconstruction. The initial calculations were done using only the multiple sequence alignment of RNase H-like domains from representative sequences. However, based on sequence data alone, we were unable to obtain a well-resolved tree. For this reason, in addition to the multiple sequence alignment, we used pairwise family similarity scores and catalytic core conservation scores (see ‘Materials and Methods’ section for details). Phylogenetic analysis based on this combined data set yielded a tree that allowed us to group reliably most of the RNHL families into 12 clades (A–F and I–VI).

#### Separation of endonucleases and exonucleases

**Figure 6.**
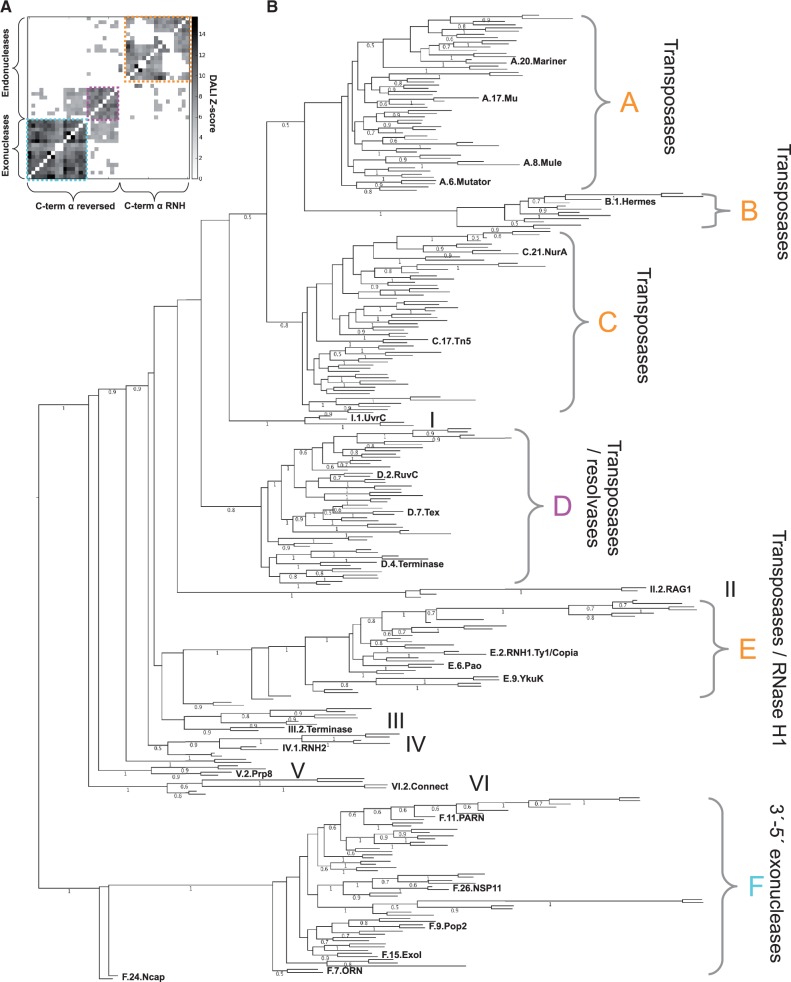
Evolutionary tree of the RNHL superfamily. (**A**) Clustering of RNHL structures based on DALI Z-scores. Clusters corresponding to 3′–5′ exonucleases and endonucleases with reversed C-terminal helix are outlined with cyan and magenta boxes, respectively. Remaining endonucleases are outlined with an orange box. (**B**) Evolutionary tree of the RNHL representatives calculated based on multiple sequence alignment, profile-profile comparisons and conservation of the catalytic residues. Main clades are indicated with letters A–F and Roman numerals I–VI. Numbers below branches indicate posterior probabilities and only those higher than 0.5 are shown.

The tree obtained based on the combined data set (Supplementary File S5) revealed clear division of the RNHL superfamily (Figure 6) into exonucleases (clade F) and endonucleases (clades A, B, C, D and E). Clade F contains almost exclusively 3′–5′ exonucleases, whose structures are characterized by a reversal of the C-terminal helix with respect to strand β1. This group includes DNA polymerases I, II, III (together with 3′ repair exonuclease 2—TREX2 and RNase T), B, Phi29, Taq and T7 and various proteins such as of ISG20, PAN2, RNA exonucleases 1, 3 and 4 (REXO1, REXO3, REXO4), Rrp6p, RNase D, PARN, Werner syndrome ATP-dependent helicase (WRN), Pop2, 3′ histone mRNA exonuclease 1 (THEX1), sporulation inhibitor (KapD), Exoribonucleases I and VIII, Arenavirus nucleocapsid and Poxvirus F12L. Uncharacterized families PF13482 (COG3359 and COG2251), PF11074 (DUF2779) and KOG2405 localized in this clade presumably also exhibit 3′–5′ exonuclease activities.

Clades A and B comprise endonucleases and are dominated by the presence of transposases and integrases. Among them we found Hermes (PDB: 2bw), Mutator, Mariner (PDB: 2f7t), Tigger, bacteriophage Mu (PDB: 1bco), MULE and IS1 transposases and several integrase families (PDB: 1qs4). Family DUF1092 (PF06485), identified in this study as new member of RNHL superfamily, was found in clade A, indicating its involvement in the transposition/integration activity. Transposases of hAT transposons DUF4371 (PF14291) and DUF659 (PF04937) were found in clade B together with P element transposase (Tnp_P_element, PF12017). A closely related clade C consists almost entirely of transposases/insertion sequences such as IS4, Tn5 (PDB: 1b7e) and ISC1217, but also includes a subclade comprising four families of archaeal and bacterial NurA, two of which are structurally characterized (PDB: 1zup and 3tai). Importantly, the NurA nucleases exhibit both a single-stranded endonuclease activity and a 5′–3′ exonuclease activity on single-stranded and double-stranded DNA (43), and are the only instances of exonucleases outside the F clade observed in our analysis. Taking into account that the architecture of NurA active site and the direction of the C-terminal α-helix are characteristic for endonucleases, we conclude that their exonuclease activity must have evolved independently from other exonucleases.

The exact branching points of clades D and E could not be resolved; however, both these clades are clearly more related to endonuclease clades (A, B and C) than to the exonuclease clade F. Clade D contains transposases (all from COG3547) and terminases (PDB: 3cpe and 3n4q), as well as resolvases (RuvC, RuvV, RuvX, Ydc2, YqgF). This clade also encompasses Tex (pdb: 3bzk), Spt6, Csn1 CRISPR-associated nuclease (COG3513), *Lactococcus* phage M3 (DUF3882/PF07066) proteins and newly identified RNHL superfamily members: DUF3010 (PF11215), DUF460 (COG2433/PF04312), RRXRR (PF14239) and DUF429 (PF04250 and COG2410, COG4328, COG4923). Interestingly, all structures present in this clade have the reversed variant of the C-terminal α-helix, which is otherwise found only in the exonuclease clade F. Clade E contains RNases H1, including representative structures 1s1x from HIV, 2g8h from *Bacillus halodurans* and 2rn2 from *Escherichia coli*. This clade also includes YkuK (PF04308), RNHL domain from Hepatitis Protein P (PF00336) and several groups of transposases and RNasesH of retroelements such as Sola, Ty1/Copia, Bel/Pao (PF05380) and archaeal COG5558. The structures present in this clade have a variant of the C-terminal α-helix characteristic for RNase H, the same as in clades A, B and C.

In the endonuclease part of the tree, we identified three smaller clades I, II and III. Clade I encompasses UvrC (2nrt) and Endonuclease V (2w36), clade II contains RAG1 protein and DUF1280 (PF06918), whereas clade III groups together phage terminases (COG5525 and COG1783), Tn3 transposase (COG4644) and a family of putative transposases DUF1258 (PF06869) that clusters together with Transposase_21 (PF02992). The branching points of the remaining small clades (IV, V and VI) could not be predicted with confidence; however, they also seem to be more related to endonucleases than to exonucleases. Clade IV contains two transposase families (represented by IS66 transposon), uncharacterized family DUF4152 (PF13680) (56) and type 2 RNase H (COG0164, COG1039, KOG2299, PF01351). Clade V groups Prp8 (PF12134, COG5178, KOG1795, PDB: 3e9l) with DUF99 (PF01949). Finally, clade VI contains RNHL domains of the mediator complex subunit 13 (PF06333, KOG3600) and Maelstrom protein (PF13017). In contrast to the clustering results, reverse transcriptase connection domain (PF06815) was also found in this clade. This, however, might be an artifact resulting from the long-branch attraction effect.

#### Evolution of the structural features

In the course of evolution, the RNHL fold was affected by several rearrangements, including the reorientation of the C-terminal helix and the emergence of an additional β-strand next to the third β-strand. Although the results of the analysis of structural features were not used directly to generate the phylogenetic tree, the correlation between the structural features and the tree topology can be observed. One important feature, already emphasized in this work, is the orientation of the C-terminal α-helix of the catalytic core (which was not included in the alignment used to generate the tree). The same orientation of this α-helix is observed in all 3′–5′ exonucleases (clade F), but also in the HJRs, Tex protein and other members of clade D. The opposite orientation of the C-terminal α-helix is observed for all other endonucleases, i.e. those from clades A, B, C and E. Because clade D is not the closest relative of exonucleases (clade F), it is plausible that RNHL domains characterized by the reversed C-terminal helix are not monophyletic and evolved independently in the two aforementioned groups. The hypothesis of multiple origins of C-terminal helix reversion is also supported by the structural data. According to DALI clustering (Figure 6), structures with reversed C-terminal helix fall into two separate clusters corresponding to clade F and clade D.

One can hypothesize that the common ancestor of RNHL superfamily was an endonuclease. In this scenario, the independent emergence of clades F and D involved change of the direction of the C-terminal α-helix with respect to strand β1. Alternatively, the ancestral RNHLs could have been 3′–5′ exonucleases from which endonucleases have evolved. In that alternative scenario, the origin of the endonuclease activity is associated with a reversion of the C-terminal helix, which has later returned to the ancestral state only in clade D.

Interestingly, the change in the orientation of the C-terminal helix in clade D correlates with the change in the active site: in contrast to RNHLs from clades A, B, C and E that usually have Glu in fourth β, the members of clade D have Asp at the equivalent position.

Another structural feature worth discussing is the presence of an additional β-strand next to the third β-strand of the catalytic core. This structural element appeared there three times independently, as it was formed by three nonhomologous sequence regions. In the structures of 3′–5′ exonucleases from clade F: 1uoc, 2fbt, 2kfn and 2a1r, that additional β-strand is formed by the N-terminal part of the RNase H-like domain, so in the sequence it is located before the first β-strand of the catalytic core. In the structure of type B polymerase, 1q8i, localized also in clade F and in the structure 1zup, localized in a distantly related clade C, analogous β-strand is formed by an insertion between the third β-strand and the first α-helix of the core. An additional β-strand in this position and with the same orientation is also present in the structure of the yeast mitochondrial HJR Ydc2 (1kcf, clade D), but in this case the element is formed by the C-terminal fragment of the protein sequence.

Also the additional β-strands (usually one, two or three) next to the fifth β-strand of the catalytic core can be of different origins. In the structures of the exonuclease domains of DNA polymerase type B (1noy and 1xhx), there are two and three additional β-strands present, respectively, formed by an insertion between the second α-helix and the fifth β-strand of the core. In case of the structures 1b7e, 1zup, 2qh9 and 2w36, the β-strands were formed by an insertion between the fifth β-strand and the C-terminal α-helix. In 2qh9, there are two β-strands present (as in 1noy), but there are also two additional α-helices. In the structures 1b7e and 1zup there are three additional β-strands, of which the first two have the same orientation and topology as in all the other structures, while the orientation of the third β-strand is opposite to the orientation of the third β-strand in 1xhx. Additional β-strands, but with different topology, are also present in the structures 2nrt and 1u04, while in the structure 1w9h the β-strands are shortened in respect to the corresponding β-strands in 1u04.

### Most common domain composition patterns

The diversity of functions and substrates in proteins containing the RNase H-like domain is reflected not only in the variability within that domain, but also in the presence and order of other domains in full-length protein sequences. To identify groups of enzymes with similar combinations of domains, we conducted additional domain and fold recognition analyses for sequence regions outside of the RNHL domain (see ‘Materials and Methods’ section for details). Representative diagrams are shown in Supplementary Figure S1. Some of the RNHL superfamily proteins possess only the core domain, typically decorated with additional α-helices and β-strands. However, in the majority of cases, the RNase H-like domain is associated with other domains, with diverse structures and functions, fused N- and/or C-terminally. In some cases, other domains are inserted into the RNase H-like domain. In the PARN, the R3H domain is inserted, which may function as a nucleic acid binding domain, while in the Hermes DNA transposases a large all α-helical domain is inserted. DNA polymerase III PolC-type is an example of a converse situation, where the RNase H-like domain is inserted into another domain. In many cases, regions of predicted intrinsic disorder are present in the terminal extensions and between the domains.

Nearly all members of the RNase H-like superfamily are nucleases; hence, they often contain domains implicated in RNA and/or DNA binding. The domains that co-occur most commonly with the RNHL domain are the different types of Helix-turn-Helix (HtH) domains, as well as different types of zinc fingers. HtH domains are present in many of the transposases and integrases, whereas zinc fingers appear to be used less frequently in these families. We identified only one transposase family that posseses both HtH and zinc finger domains: COG3676 (PF12762), e.g. GI: 4467436 from *Halobacterium salinarum* and GI: 375358235 from *Bacteroides fragilis*. In the context of transposases/integrases we detected also other domains, for instance TnsA-like endonuclease domain (PF08722+PF08721) was found in Mu-like transposon GI: 383757636 from *Rubrivivax gelatinosus* (family A.14). Many of those domains that do not have experimentally assigned functions (DUFs) were found to be distant relatives of HtH motifs (e.g. PF14210 co-occurring with C.19 and PF05598 co-occurring with C.2, C.7, C.11, C.12, C.14 and C.20). For others, like PF14294 (co-occurring with C.14 and C.28), PF13006 (C.15 and C.28), PF13808 (C.9) and PF13700 (III.3), we could not identify evident homologs with known structures and functions; however, they probably also adopt a HtH fold.

Many proteins contain more than one copy of an RNHL domain. The most typical arrangement, characteristic for the *pol* polyporotein of LTR retrotransposons and retroviruses, is a tandem array of RNase H 1-like from clade E and integrase domain from clade A. In the case of Ty1/Copia transposons, the order of the two domains is reversed, i.e. integrase domain (PF00665) occurs N-terminally to RNase H 1-like domain (PF00075). In animal retroviruses the above-mentioned array is preceded by another RNHL domain (57). For example, HIV *pol* protein (GI: 55740237) is characterized by the presence of a connection (PF06815, family VI.2), RNase H (PF00075, family E.8) and integrase (PF00665, family A.2) domains.

Some domains are specific for particular families or clades. For example, the hAT dimerization domain (PF05699) is frequently present in the context of RNHL domains from clade B, i.e. B.2 (DUF4371; PF14291), B.3 (DUF659; PF04937), B.5 and in Activator-like transposases B.1 and B.6 (KOG1121). The only families in clade B, whose members do not contain hAT domain, are P elements with a Tnp_P_element domain (PF12017) (58). Other examples of family-specific domain architectures are the UvrC proteins that comprise RNase H-like domain, GIY-YIG nuclease domain, UvrB binding domain (UvrBb), specific region containing four conserved cysteines (CCCC) and a DNA binding domain (HhH) (59). Also the Piwi/Argonaute nucleases represent specific domain architecture, dissimilar to all other families, characterized by an N-terminal domain, a PAZ domain, a Middle domain and a Piwi domain with the RNase H-like fold.

Newly identified RNHL families are frequently characterized by specific and conserved domain composition patterns. For instance, in RRXRR proteins (PF14239) localized in family D.10, the RNHL domain is frequently accompanied C-terminally by the HNH nuclease domain. This architecture resembles the CRISPR-associated nucleases (COG3513) from family D.16. Most members of DUF429 (clade D) family are single-domain proteins with a few exceptions co-occuring with a NUDIX hydrolase domain (in Alphaproteobacteria, Clostridia and Spirochaetes) or a RelA/SpoT domain (in Actinobacteria). Members of DUF3010 (PF11215, family D.19) comprise a single RNHL domain. In COG2433 (PF04312, DUF460, family D.18) also only the RNHL domain was detected; however, the average length of proteins in this family suggests presence of other domains.

Family F.16 (KOG2405; PF01612) from the exonuclease clade F co-occurs with the Helicase-and-RNase-D C-terminal domain. This domain is also present in two other families within clade F: F.6 (KOG4373/KOG2207; PF01612) and F.4 (KOG2206/COG0349; PF01612) families represented by WRN exonuclease and RNase D, respectively. RNHL domains in proteins from COG3359 (PF13482, family F.10) are frequently followed by Tetratricopeptide repeats. Families F.17 (DUF2779; PF11074) and F.13 (COG2251; PF13482) were found to contain DUF83, which resembles PD-(D/E)XK nucleases. In COG2251, a tandem repeat of AAA domains is frequently observed. Some members of family F.8 (COG5018/KOG0542; PF00929) co-occur with RNA recognition motif domains, for example, GI: 254572319 from *Komagataella pastoris*.

As already mentioned, many of the families comprise only the RNHL domain (see also Supplementary Figure S1), for example, A/26 (DUF1092; PF06485), D.19 (DUF3010; PF11215), IV.3 (DUF4152; PF13680), as well as E.9 (DUF458/COG1978; PF04308), F.28 (PF03337). However, one should keep in mind that the PFAM annotations can be misleading, for instance, PF05876 is not a single domain but corresponds to RNHL and P-loop NTPase domains.

## SUMMARY

In this work, we carried out a comprehensive comparative analysis of the RNHL superfamily, for the first time including representatives of all its known families. In a multi-step search of structure and sequence databases we managed to identify >60 000 member proteins of the RNHL superfamily, including proteins previously unknown to be homologous to RNase H. We conducted clustering of the collected sequences and based on their similarity we divided them into groups of most closely related proteins. We also conducted an extensive analysis of sequence conservation and structural features of representatives of separated families and subfamilies, and we inferred a multiple sequence alignment for the conserved core region. Based on comparison of sequences and structural features we calculated a phylogenetic tree of the superfamily, which revealed deep evolutionary relationships between strongly diverged branches comprising various nucleases, and proteins without known catalytic activities. One of the most striking observations is a clear division of RNase H-like domains of endonucleases and 3′–5′ exonucleases. We can observe significant differences between these two groups in all analyzed aspects. The differences of the architecture of the RNase H-like domain entail the differences in the architecture of the active site, including the position and identity of catalytic residues, position of the catalytic metal ions as well as the position of the substrate nucleic acid with respect to the RNHL fold. Our analysis also reports the identification of new RNHL superfamily members, such as RRXRR (PF14239), DUF460 (PF04312, COG2433), DUF3010 (PF11215), DUF429 (PF04250 and COG2410, COG4328, COG4923), DUF1092 (PF06485), COG5558, OrfB_IS605 (PF01385, COG0675) and Peptidase_A17 (PF05380). Altogether, our study presents a comprehensive picture of sequence-structure-function relationships among RNHL superfamily members. We hope this analysis will help to understand relationships between proteins that have been functionally characterized, and to predict functions and plan experiments for proteins that have not yet been studied.

## SUPPLEMENTARY DATA

Supplementary Data are available at NAR Online.

## FUNDING

European Research Council (ERC) [StG RNA + P = 123D to J.M.B.]; Foundation for Polish Science [FNP, TEAM/2010-6 to K.G.], Polish National Science Centre [NCN, 2011/02/A/NZ2/00014 to K.G.]; statutory funds of the Adam Mickiewicz University (to K.A.M., in part); Ministry of Science and Higher Education [grant Iuventus Plus 0376/IP1/2011/71 to A.M.]; European Social Fund [UDA-POKL.04.01.01-00-072/09-00 to K.S.]; International Early Career Scientist grant from the Howard Hughes Medical Institute (to M.N., in part); ‘Ideas for Poland’ fellowships from the FNP (to M.N. and J.M.B.) and ‘START’ fellowship from the FNP (to S.D.H.). Funding for open access charge: ERC [StG grant RNA + P = 123D].

*Conflict of interest statement*. None declared.

## Supplementary Material

Supplementary Data
