# Supplementary material for: The RNase H-like superfamily: new members, comparative structural analysis and evolutionary classification
Source: Nucleic Acids Res. 2014 Jan 23;42(7):4160–79. doi: 10.1093/nar/gkt1414 (PMC3985635; doi:10.1093/nar/gkt1414)
Supplement: Supplementary Data [file supp_42_7_4160__index.html]

The RNase H-like superfamily: new members, comparative structural analysis and evolutionary classification — The RNase H-like superfamily: new members, comparative structural analysis and evolutionary classification — Supplementary Data 

# The RNase H-like superfamily: new members, comparative structural analysis and evolutionary classification

## Supplementary Data

files

**Files in this Data Supplement:**

- Supplementary Data - zip file
