# Supplementary material for: The RNase H-like superfamily: new members, comparative structural analysis and evolutionary classification
Source: Nucleic Acids Res. 2014 Jan 23;42(7):4160–79. doi: 10.1093/nar/gkt1414 (PMC3985635; doi:10.1093/nar/gkt1414)
Supplement: Supplementary Data [file supp_gkt1414_nar-02849-r-2013-File008.zip › SupplementaryFigure1.pdf]

FAMILY A.6.Mutator.PF00872.PF10551.COG3328

gi|81458892|sp|Q8E9J3|Q8E9J3_SHEON ISSod5, transposase

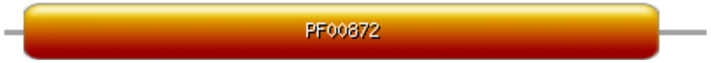

PF00872 - Transposase, Mutator family

FAMILY A.2.PF00665

gi|55740237|gb|AAV63810.1| PF00665 pol protein [Human immunodeficiency virus 1]

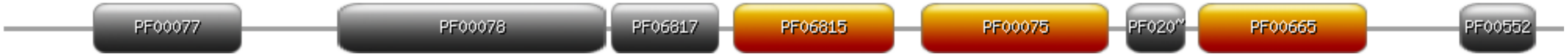

PF00077 - Retroviral aspartyl protease

PF06815 - Reverse transcriptase connection domain

PF00665 - Integrase core domain

PF00078 - Reverse transcriptase (RNA-dependent DNA polymerase)

PF00075 - RNase H

PF06817 - Reverse transcriptase thumb domain

PF00552 - Integrase DNA binding domain

PF02022 - Integrase Zinc binding domain

FAMILY A.4.PF03184.KOG3105

gi|392573028|gb|EIW66170.1| hypothetical protein TREMEDRAFT_35394 [Tremella mesenterica DSM 1558]

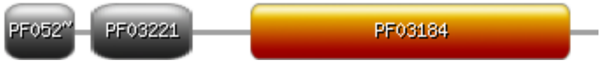

gi|74762678|sp|Q96MW7.1|TIGD1_HUMAN RecName: Full=Tigger transposable element-derived protein 1

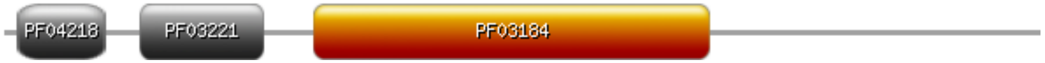

PF03184 - DDE superfamily endonuclease

PF03221 - Tc5 transposase DNA-binding domain

PF05225 - helix-turn-helix, Psq domain

PF04218 - CENP-B N-terminal DNA-binding domain

FAMILY A.20.PF01359

gi|308482157|ref|XP_003103282.1| hypothetical protein CRE_27688 [Caenorhabditis remanei]

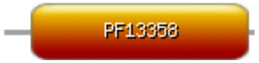

gi|74959823|sp|061446|061446_DROSE Transposase

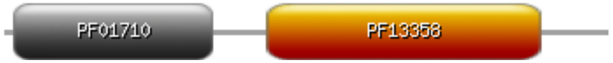

PF13358 - DDE superfamily endonuclease

PF01710 - Transposase

FAMILY A.17.PF02914.PF00665.COG2801

gi|254510363|ref|ZP_05122430.1| PF02914 integrase, catalytic region [Rhodobacteraceae bacterium KLH11]

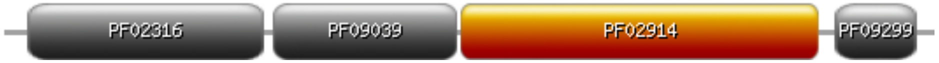

PF02914 - Bacteriophage Mu transposase

PF02316 - Mu DNA-binding domain

PF09039 - Mu DNA binding, I gamma subdomain

PF09299 - Mu transposase, C-terminal

FAMILY A.8.Mule.PF10551

gi|123472691|ref|XP_001319538.1| hypothetical protein [Trichomonas vaginalis G3]

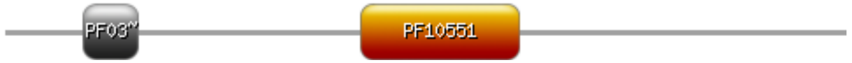

gi|116310978|emb|CAH67914.1| PF10551 OSIGBa0115K01-H0319F09.20 [Oryza sativa Indica Group]

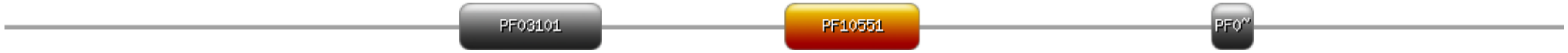

PF10551 - MULE transposase domain

PF03101 - FAR1 DNA-binding domain

PF04434 - SWIM zinc finger

FAMILY A.22.PF03400.COG1662

gi|237809941|ref|YP_002894380.1| InsB1 [Escherichia coli]

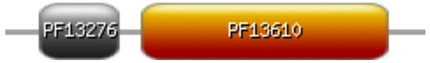

gi|340622954|ref|YP_004741406.1| insertion element iS1 4 protein insB [Capnocytophaga canimorsus Cc5]

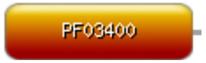

PF13610 - DDE domain

PF13276 - HTH-like domain

PF03400 - IS1 transposase

FAMILY A.25.PF00665

gi|77463343|ref|YP_352847.1| transposase [Rhodobacter sphaeroides 2.4.1]

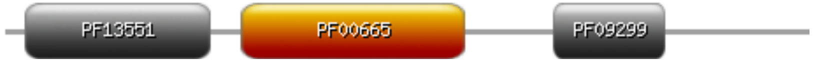

PF00665 - Integrase core domain

PF13551 - Winged helix-turn helix

PF09299 - Mu transposase, C-terminal

FAMILY A.24.PF07592

gi|81659280|sp|Q7UJR7|Q7UJR7_RHOBA Similar to transposase

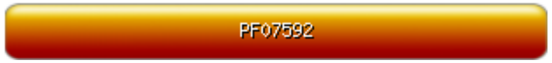

gi|373482322|ref|ZP_09573131.1| hypothetical protein SinacDRAFT_0705 [Singulisphaera acidiphila DSM 18658]

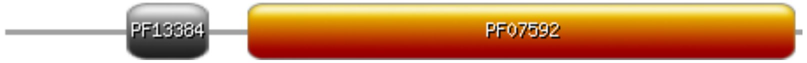

PF07592 - Rhodopirellula transposase DDE domain

PF13384 - Homeodomain-like domain

FAMILY A.7.PF00665.COG4584

gi|377561901|ref|ZP_09791327.1| transposase [Gordonia otitidis NBRC 100426]

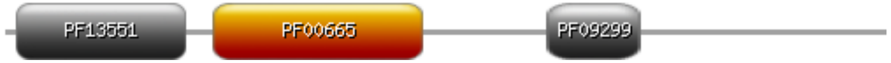

PF00665 - Integrase core domain

PF13551 - Winged helix-turn helix

PF09299 - Mu transposase, C-terminal

FAMILY A.12.PF00665.COG2801

gi|122245631|sp|Q33AB6|Q33AB6_ORYSA Retrotransposon protein, putative, Ty3-gypsy subclass

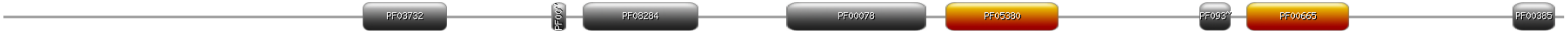

PF08284 - Retroviral aspartyl protease

PF00665 - Integrase core domain

PF00078 - Reverse transcriptase (RNA-dependent DNA polymerase)

PF03732 - Retrotransposon gag protein

PF09337 - His(2)-Cys(2) zinc finger

PF05380 - Pao retrotransposon peptidase

PF00385 - Chromo (CHRromatin Organisation MOdifier) domain

PF00098 - Zinc knuckle

FAMILY A.3.PF13358.COG3335

gi|119947277|ref|YP_944957.1| ISSod10, transposase OrfB [Psychromonas ingrahamii 37]

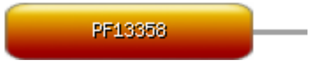

gi|75440988|sp|Q6XGE3|Q6XGE3_ECOLI TnpA-like protein

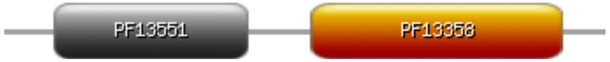

PF13358 - DDE superfamily endonuclease

PF13551 - Winged helix-turn helix

FAMILY A.10.PF13358

gi|141446|sp|P03934.1|TC1A_CAEEL RecName: Full=Transposable element Tc1 transposase

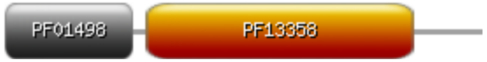

gi|332027221|gb|EGI67310.1| Transposable element Tcb1 transposase [Acromyrmex echinatior]

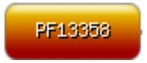

gi|221107703|ref|XP_002156451.1| PREDICTED: similar to transposase-like [Hydra magnipapillata]

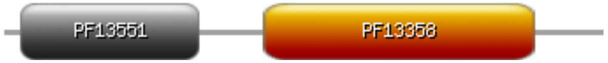

PF13358 - DDE superfamily endonuclease

PF01498 - Transposase

PF13551 - Winged helix-turn helix

FAMILY A.16.PF13610.PF03050.COG3316

gi|196188062|gb|EDX83027.1| hypothetical protein S7335_205 [Synechococcus sp. PCC 7335]

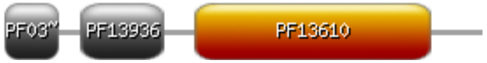

gi|81775735|sp|Q981F5|Q981F5_RHILO Transposase

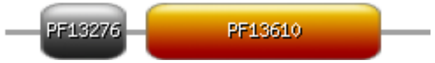

gi|228995360|ref|ZP_04155045.1| PF13610 hypothetical protein bpmyx0001_59630 [Bacillus pseudomycoides DSM 12442]

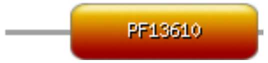

PF13610 - DDE domain

PF03811 - InsA N-terminal domain

PF13936 - Helix-turn-helix domain

PF13276 - HTH-like domain

FAMILY A.13.PF12762.COG3676

gi|198411948|ref|XP_002129449.1| PREDICTED: hypothetical protein [Ciona intestinalis]

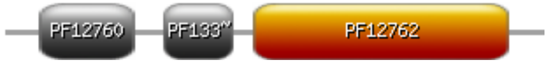

gi|375358235|ref|YP_005111007.1| PF12762 putative transposase [Bacteroides fragilis 638R]

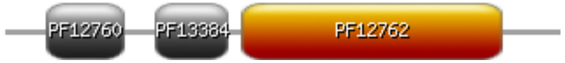

gi|4467436|emb|CAB37934.1| hypothetical protein [Halobacterium salinarum]

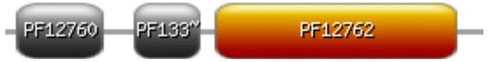

PF12762 - ISXO2-like transposase domain

PF12760 - Transposase zinc-ribbon domain

PF13384 - Homeodomain-like domain

FAMILY A.28

gi|340386950|ref|XP_003391971.1| PREDICTED: hypothetical protein LOC100633410, partial [Amphimedon queenslandica]

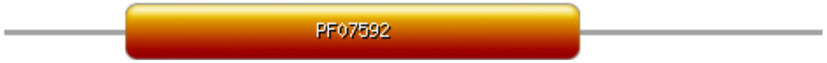

PF07592 - Rhodopirellula transposase DDE domain

FAMILY A.9.PF01610.COG3464

gi|226349911|ref|YP_002777024.1| putative transposase for insertion sequence element [Rhodococcus opacus B4]

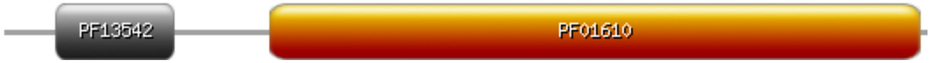

gi|549101|sp|Q06126.1|TNPA_BORPP RecName: Full=Transposase for insertion sequence element IS1001

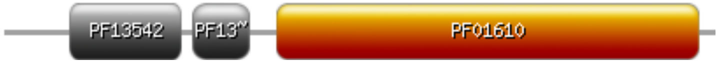

PF01610 - Transposase

PF13542 - Helix-turn-helix domain of transposase family ISL3

PF13384 - Homeodomain-like domain

FAMILY A.5.PF00665

gi|75140134|sp|Q75IE4|Q75IE4_ORYSA Putative Gag and Pol polyprotein

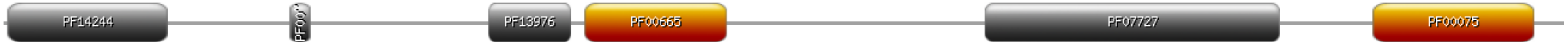

PF07727 - Reverse transcriptase (RNA-dependent DNA polymerase)

PF14244 - gag-polypeptide of LTR copia-type

PF00665 - Integrase core domain

PF13976 - GAG-pre-integrase domain

PF00098 - Zinc knuckle

PF00075 - RNase H

FAMILY A.18.PF00665.COG2801

gi|269468916|gb|EEZ80503.1| PF00665 transposase [uncultured SUP05 cluster bacterium]

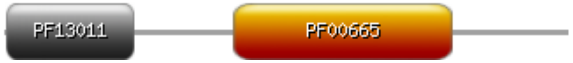

gi|84497088|ref|ZP_00995910.1| PF00665 PF13683 transposase [Janibacter sp. HTCC2649]

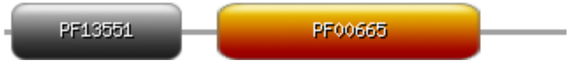

PF00665 - Integrase core domain

PF13011 - leucine-zipper of insertion element IS481

PF13551 - Winged helix-turn helix

FAMILY A.27.PF00665

gi|383784580|ref|YP_005469150.1| putative integrase, catalytic subunit [Leptospirillum ferrooxidans C2-3]

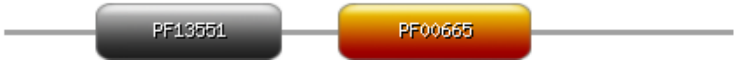

PF00665 - Integrase core domain

PF13551 - Winged helix-turn helix

FAMILY A.11.PF00665.COG2826

gi|209694648|ref|YP_002262576.1| PF00665 transposase [Aliivibrio salmonicida LFI1238]

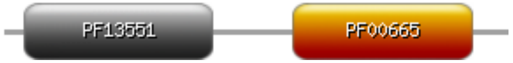

gi|296439650|sp|P0CF88.1|INSI1_ECOLI RecName: Full=Transposase InsI for insertion sequence element IS30A

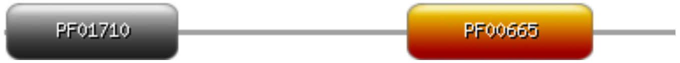

PF00665 - Integrase core domain

PF13551 - Winged helix-turn helix

PF01710 - Transposase

FAMILY A.14.PF00665.COG2801

gi|383757636|ref|YP_005436621.1| integrase catalytic subunit [Rubrivivax gelatinosus IL144]

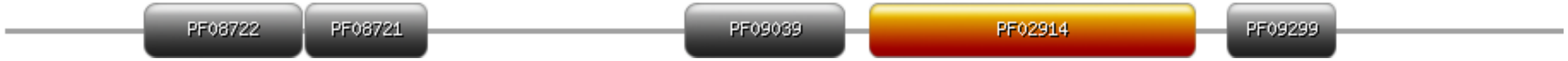

gi|135965|sp|P13989.1|TNSB_ECOLX RecName: Full=Transposon Tn7 transposition protein TnsB

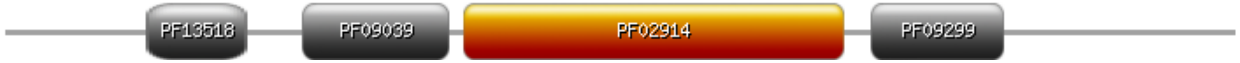

gi|194367340|ref|YP_002029950.1| integrase catalytic subunit [Stenotrophomonas maltophilia R551-3]

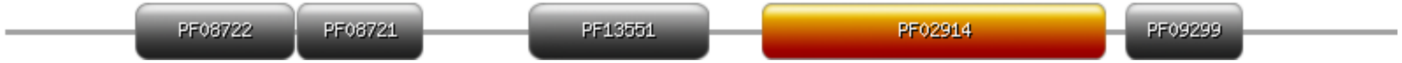

PF02914 - Bacteriophage Mu transposase

PF08722 - TnsA endonuclease N terminal

PF09299 - Mu transposase, C-terminal

PF09039 - Mu DNA binding, I gamma subdomain

PF08721 - TnsA endonuclease C terminal

PF13518 - Helix-turn-helix domain

PF13551 - Winged helix-turn helix

FAMILY A.19.PF00665.PF13683.COG2801

gi|294956555|sp|P0CF53.1|INSD1_ECOLI RecName: Full=Transposase InsD for insertion element IS2A

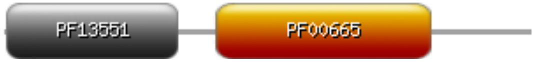

PF00665 - Integrase core domain

PF13551 - Winged helix-turn helix

FAMILY A.1.PF00665.PF13333.COG2801

gi|241889773|ref|ZP_04777071.1| PF00665 putative transposase InsK for insertion sequence [Gemella haemolysans ATCC 10379]

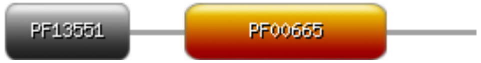

gi|75442125|sp|Q75TV8|Q75TV8_BACHD Transposase B of IS655

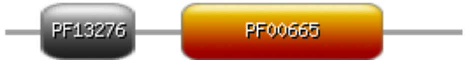

PF00665 - Integrase core domain

PF13551 - Winged helix-turn helix

PF13276 - HTH-like domain

FAMILY A.15.PF13683.COG2801

gi|94495266|ref|ZP_01301847.1| transposase orfB [Sphingomonas sp. SKA58]

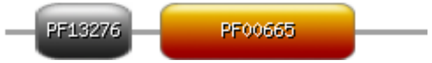

gi|205371837|sp|P24577.2|YI71_BURM1 RecName: Full=Insertion element IS407 uncharacterized 31.7 kDa protein; AltName: Full=ORF1

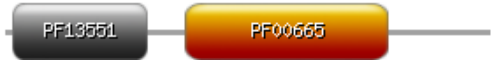

PF00665 - Integrase core domain

PF13276 - HTH-like domain

PF13551 - Winged helix-turn helix

FAMILY A.21.PF00665

gi|254587304|emb|CAX83709.1| PF00665 Gag-Pol polyprotein [Schistosoma japonicum]

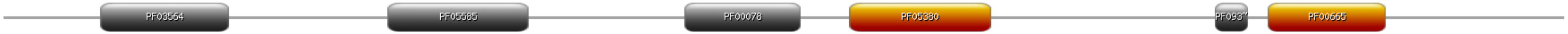

PF05380 - Pao retrotransposon peptidase

PF03564 - Protein of unknown function (DUF1759)

PF05585 - Putative peptidase (DUF1758)

PF00665 - Integrase core domain

PF00078 - Reverse transcriptase (RNA-dependent DNA polymerase)

PF09337 - His(2)-Cys(2) zinc finger

FAMILY A.26.DUF1092.PF06485

gi|75906361|ref|YP_320657.1| PF06485 hypothetical protein [Anabaena variabilis ATCC 29413]

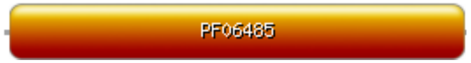

PF06485 - Protein of unknown function (DUF1092)

FAMILY A.23.PF06782

gi|29839719|sp|Q8KRA0.1|YVAB_STRGY RecName: Full=UPF0236 protein in vanSb 3'region

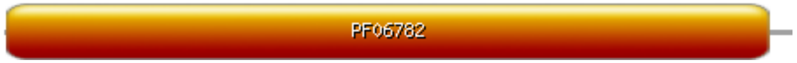

PF06782 - Uncharacterised protein family (UPF0236)

FAMILY B.2.DUF4371.PF14291

gi|12643558|sp|O43422.2|P52K_HUMAN RecName: Full=52 kDa repressor of the inhibitor of the protein kinase; Short=p52rIPK; AltName: Full=58 kDa interferon-induced protein kinase-interacting protein; Short=p58IPK-interacting protein; AltName: Full=Death-associated protein 4; AltName: Full=THAP domain-containing protein 0

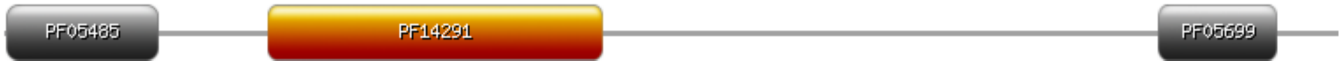

PF14291 - Domain of unknown function (DUF4371)

PF05485 - THAP domain

PF05699 - hAT family dimerisation domain

FAMILY B.5

gi|170027752|ref|XP_001841761.1| p20-CGGBP [Culex quinquefasciatus]

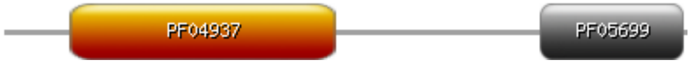

gi|391333164|ref|XP_003740991.1| PREDICTED: uncharacterized protein LOC100904406 [Metaseiulus occidentalis]

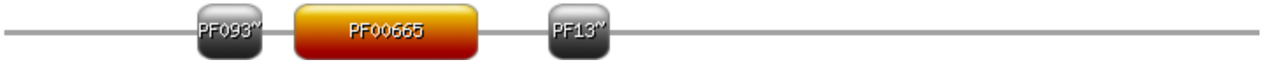

PF04937 - Protein of unknown function (DUF 659)

PF05699 - hAT family dimerisation domain

PF00665 - Integrase core domain

PF09337 - His(2)-Cys(2) zinc finger

PF13923 - Zinc finger, C3HC4 type (RING finger)

FAMILY B.3.DUF659.PF04937

gi|359483259|ref|XP_002269962.2| PREDICTED: uncharacterized protein LOC100251332 [Vitis vinifera]

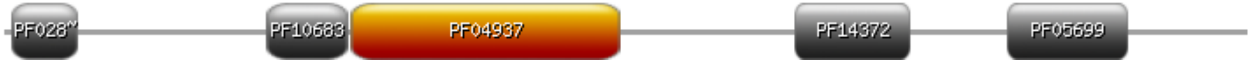

PF04937 - Protein of unknown function (DUF 659)

PF05699 - hAT family dimerisation domain

PF02892 - BED zinc finger

PF14372 - Domain of unknown function (DUF4413)

PF10683 - Hermes transposase DNA-binding domain

FAMILY B.1.Hermes.PF04937.KOG1121

gi|322702335|gb|EFY94003.1| putative reverse transcriptase [Metarhizium anisopliae ARSEF 23]

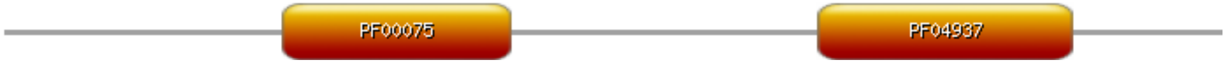

gi|74766139|sp|Q25438|Q25438_MUSDO Hermes transposase

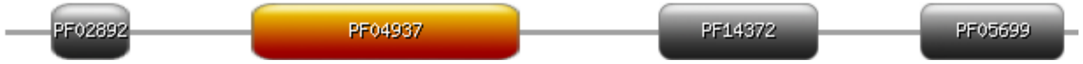

PF00075 - RNase H

PF04937 - Protein of unknown function (DUF 659)

PF05699 - hAT family dimerisation domain

PF14372 - Domain of unknown function (DUF4413)

PF02892 - BED zinc finger

FAMILY B.6.KOG1121

gi|17570705|ref|NP_510586.1| Protein ZK662.5 [Caenorhabditis elegans]

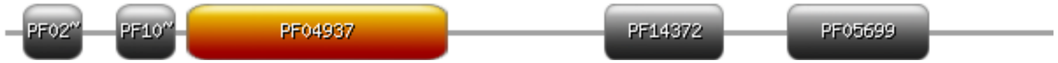

PF05699 - hAT family dimerisation domain

PF04937 - Protein of unknown function (DUF 659)

PF14372 - Domain of unknown function (DUF4413)

PF02892 - BED zinc finger

PF10683 - Hermes transposase DNA-binding domain

FAMILY B.4.PF12017

gi|88926256|sp|Q7M3K2.2|PELET_DROME PF12017 RecName: Full=Transposable element P transposase; Short=P-element transposase

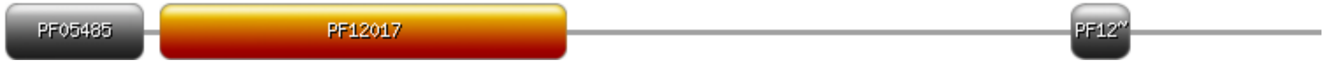

PF12017 - Transposase protein

PF05485 - THAP domain

PF12596 - 87kDa Transposase

FAMILY C.19.PF01609.COG3385

gi|16120204|ref|NP_395792.1| hypothetical protein VNG6290H [Halobacterium sp. NRC-1]

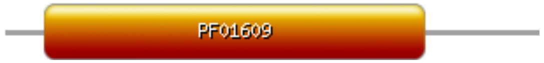

gi|229583081|ref|YP_002841480.1| transposase IS4 family protein [Sulfolobus islandicus Y.N.15.51]

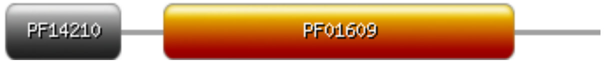

PF01609 - Transposase DDE domain

PF14210 - Domain of unknown function (DUF4322)

FAMILY C.8.PF13843

gi|307196437|gb|EFN78017.1| PiggyBac transposable element-derived protein 4 [Harpegnathos saltator]

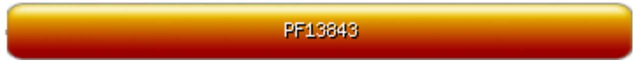

gi|221123725|ref|XP_002162345.1| PREDICTED: similar to piggyBac transposase Uribo1 [Hydra magnipapillata]

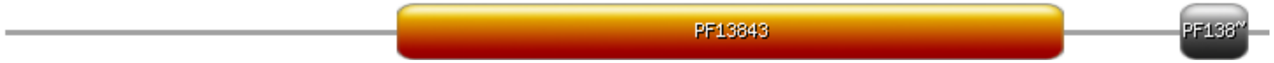

PF13843 - Transposase IS4

PF13842 - DDE_Tnp_1-like zinc-ribbon

FAMILY C.10.PF13546.COG5659

gi|386836247|ref|YP_006241305.1| putative transposase [Streptomyces hygroscopicus subsp. jinggangensis 5008]

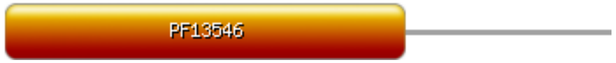

PF13546 - DDE superfamily endonuclease

FAMILY C.20.PF01609

gi|383319937|ref|YP_005380778.1| tnp-12 gene product [Methanocella conradii HZ254]

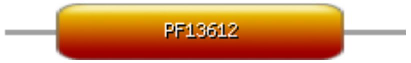

gi|47076660|dbj|BAD18242.1| transposase of IS662 [Bacillus halodurans]

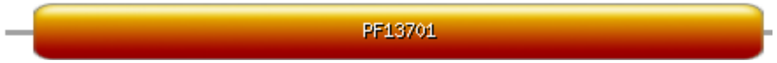

gi|326389903|ref|ZP_08211466.1| transposase IS4 family protein [Thermoanaerobacter ethanolicus JW 200]

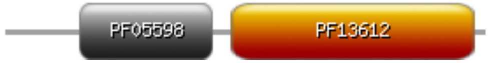

PF13612 - Transposase DDE domain

PF13701 - Transposase DDE domain group 1

PF05598 - Transposase domain (DUF772)

FAMILY C.12.PF01609.PF13737.COG3039

gi|15899730|ref|NP_344335.1| PF01609 second ORF in transposase ISC1058 [Sulfolobus solfataricus P2]

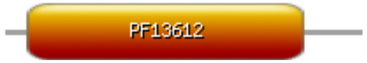

gi|136129|sp|P03009.1|TRA2_ECOLX RecName: Full=Probable transposase for transposon Tn903

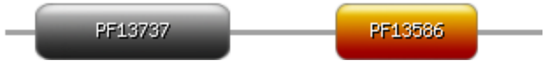

gi|340030471|ref|ZP_08666534.1| transposase, IS4 family protein [Paracoccus sp. TRP]

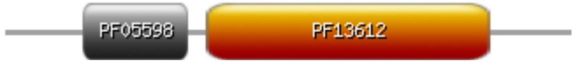

PF13612 - Transposase DDE domain

PF13737 - Transposase DDE domain

PF13586 - Transposase DDE domain

PF05598 - Transposase domain (DUF772)

FAMILY C.2.PF13751.PF1609.COG3039

gi|242138353|gb|EES24755.1| transposase, IS4 family [Burkholderia pseudomallei 1106b]

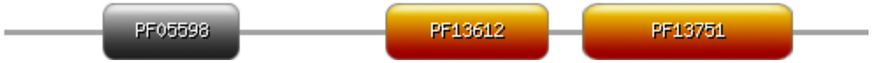

PF13751 - Transposase DDE domain

PF13612 - Transposase DDE domain

PF05598 - Transposase domain (DUF772)

FAMILY C.7.PF01609.PF13751.COG3039

gi|226944557|ref|YP_002799630.1| PF01609 transposase, IS4 [Azotobacter vinelandii DJ]

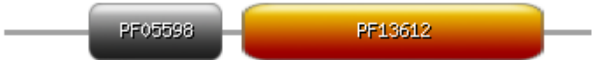

PF13612 - Transposase DDE domain

PF05598 - Transposase domain (DUF772)

FAMILY C.6.PF01609.PF13586.COG3293

gi|380344364|gb|EIA32710.1| PF01609 transposase is4 family protein [Lactobacillus salivarius SMXD51]

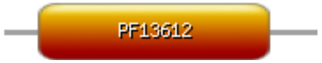

gi|122611732|sp|Q3L240|Q3L240_RHITR Transposase

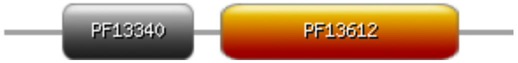

PF13612 - Transposase DDE domain

PF13340 - Putative transposase of IS4/5 family (DUF4096)

FAMILY C.4.PF13613.PF13359.KOG1121

gi|763133|emb|CAA54546.1| PF01609 PF13613 mobile genetic element [Lactobacillus helveticus]

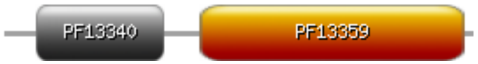

gi|386837545|ref|YP_006242603.1| transposase [Streptomyces hygroscopicus subsp. jinggangensis 5008]

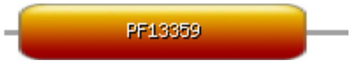

PF13359 - DDE superfamily endonuclease

PF13340 - Putative transposase of IS4/5 family (DUF4096)

FAMILY C.3.PF01609.PF13586.COG3293

gi|385339131|ref|YP_005893003.1| PF01609 transposase IS4 family [Neisseria meningitidis G2136]

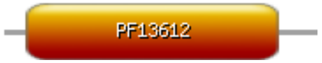

gi|122500378|sp|Q2A241|Q2A241_FRATH Transposase

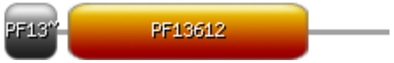

PF13612 - Transposase DDE domain

PF13340 - Putative transposase of IS4/5 family (DUF4096)

FAMILY C.11.PF01609.PF13612

gi|341631216|gb|EGS56140.1| transposase DDE domain protein [Vibrio cholerae HE-09]

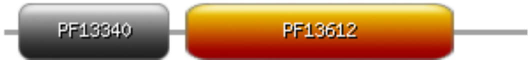

gi|75486082|sp|Q9X6G5|Q9X6G5_ENTFC Transposase

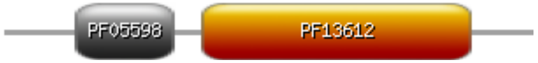

gi|380692824|ref|ZP_09857683.1| transposase_11 DDE family protein [Bacteroides faecis MAJ27]

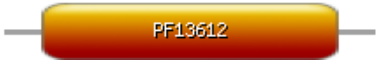

PF13612 - Transposase DDE domain

PF13340 - Putative transposase of IS4/5 family (DUF4096)

PF05598 - Transposase domain (DUF772)

FAMILY C.1.PF04827.PF13359.KOG4585

gi|294461638|gb|ADE76379.1| PF13359 unknown [Picea sitchensis]

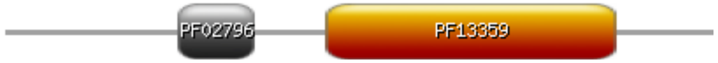

gi|328702684|ref|XP_003241982.1| PREDICTED: putative nuclease HARBI1-like [Acyrthosiphon pisum]

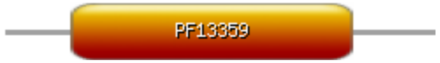

PF13359 - DDE superfamily endonuclease

PF02796 - Helix-turn-helix domain of resolvase

FAMILY C.17.PF01609

gi|75539327|sp|Q46731.1|TN5P_ECOLX RecName: Full=Transposase for transposon Tn5; AltName: Full=Tnp

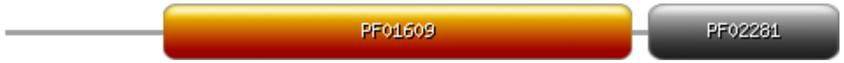

PF02281 - Transposase Tn5 dimerisation domain

PF01609 - Transposase DDE domain

FAMILY C.25.PF01609

gi|325282707|ref|YP_004255248.1| transposase IS4 family protein [Deinococcus proteolyticus MRP]

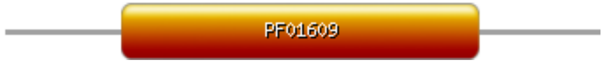

PF01609 - Transposase DDE domain

FAMILY C.13.PF13701.PF01609

gi|123452517|sp|Q1QKN3|Q1QKN3_NITHX Transposase, IS4

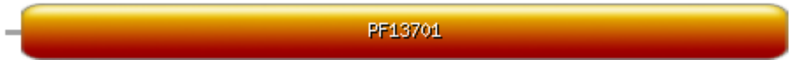

PF13701 - Transposase DDE domain group 1

FAMILY C.29.PF04693

gi|374634235|ref|ZP_09706600.1| Archaeal putative transposase ISC1217 [Metallosphaera yellowstonensis MK1]

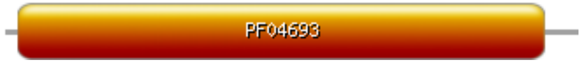

PF04693 - Archaeal putative transposase ISC1217

FAMILY C.16.PF01609.PF13546

gi|168698207|ref|ZP_02730484.1| transposase IS4 family protein [Gemmata obscuriglobus UQM 2246]

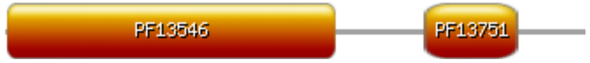

gi|75542468|sp|Q895E8|Q895E8_CLOTE Hypothetical protein

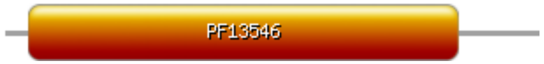

PF13546 - DDE superfamily endonuclease

PF13751 - Transposase DDE domain

FAMILY C.5.PF01609.COG5421

gi|81325552|sp|Q9ZAA3|Q9ZAA3_MYCMS Putative transposase

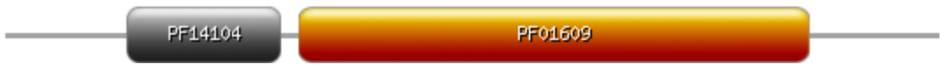

PF01609 - Transposase DDE domain

PF14104 - Domain of unknown function (DUF4277)

FAMILY C.14.PF01609.COG3385

gi|389578810|ref|ZP_10168837.1| transposase family protein [Desulfobacter postgatei 2ac9]

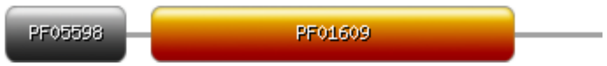

gi|81324981|sp|Q9LC69|Q9LC69_BACSU Transposase

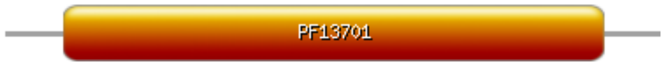

gi|308272316|emb|CBX28922.1| PF01609 hypothetical protein N47_B20680 [uncultured Desulfobacterium sp.]

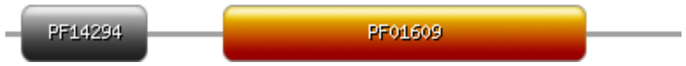

PF01609 - Transposase DDE domain

PF05598 - Transposase domain (DUF772)

PF13701 - Transposase DDE domain group 1

PF14294 - Domain of unknown function (DUF4372)

FAMILY C.28.PF01609.COG3385

gi|296439653|sp|P0CF91.1|INSL1_ECOLI RecName: Full=Putative transposase InsL for insertion sequence element IS186A

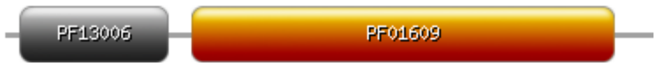

gi|329908699|ref|ZP_08274937.1| PF01609 Transposase [Oxalobacteraceae bacterium IMCC9480]

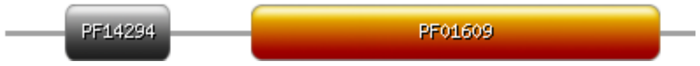

PF01609 - Transposase DDE domain

PF13006 - Insertion element 4 transposase N-terminal

PF14294 - Domain of unknown function (DUF4372)

FAMILY C.24.PF01609.PF13546.COG3385

gi|81859013|sp|Q9RZM2|Q9RZM2_DEIRA Transposase, putative

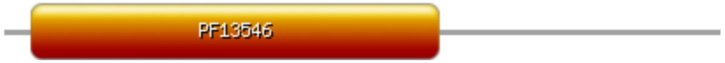

PF13546 - DDE superfamily endonuclease

FAMILY C.15.PF01609

gi|344339535|ref|ZP_08770464.1| transposase IS4 family protein [Thiocapsa marina 5811]

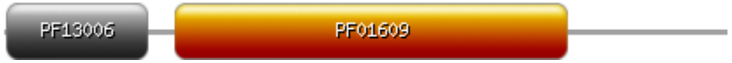

PF01609 - Transposase DDE domain

PF13006 - Insertion element 4 transposase N-terminal

FAMILY C.9.PF01609.COG5433

gi|304284345|gb|EFL92738.1| transposase [Candidatus Regiella insecticola LSR1]

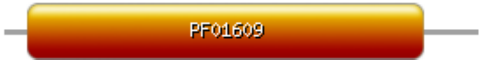

gi|140772|sp|P28912.1|YHHI_ECOLI RecName: Full=H repeat-associated protein YhhI; AltName: Full=ORF-H

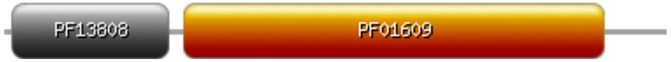

PF01609 - Transposase DDE domain

PF13808 - DDE_Tnp_1-associated

FAMILY C.26.PF13359

gi|348685031|gb|EGZ24846.1| hypothetical protein PHYSODRAFT_485494 [Phytophthora sojae]

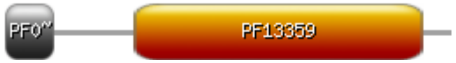

PF13359 - DDE superfamily endonuclease

PF02796 - Helix-turn-helix domain of resolvase

FAMILY C.23.PF13546

gi|297550311|gb|EFH84177.1| conserved hypothetical protein [Ktedonobacter racemifer DSM 44963]

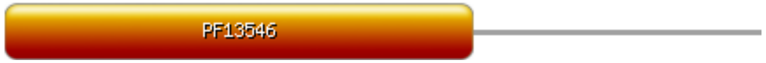

PF13546 - DDE superfamily endonuclease

FAMILY C.21.NurA.PF09376.COG2380

gi|288817580|ref|YP_003431927.1| PF09376 hypothetical protein HTH_0259 [Hydrogenobacter thermophilus TK-6]

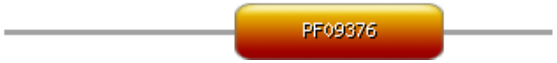

PF09376 - NurA domain

FAMILY C.27.NurA.PF09376.COG1630

gi|48477314|ref|YP_023020.1| PF09376 hypothetical protein PTO0242 [Picrophilus torridus DSM 9790]

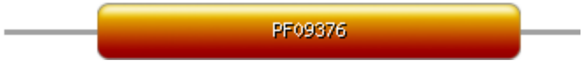

PF09376 - NurA domain

FAMILY C.18.NurA.PF09376.COG1630

gi|389852559|ref|YP_006354793.1| hypothetical protein Py04_1144 [Pyrococcus sp. ST04]

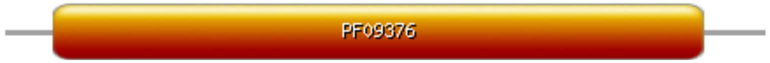

PF09376 - NurA domain

FAMILY C.22.NurA.PF09376

gi|288931955|ref|YP_003436015.1| NurA domain protein [Ferroglobus placidus DSM 10642]

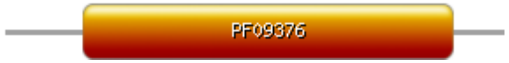

PF09376 - NurA domain

FAMILY I.1.UvrC.PF04859.COG0322

gi|149135311|gb|EDM23791.1| excinuclease ABC subunit C [Caminibacter mediatlanticus TB-2]

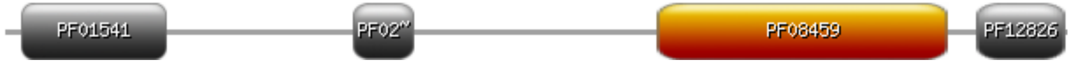

PF08459 - UvrC Helix-hairpin-helix N-terminal

PF12826 - Helix-hairpin-helix motif

PF01541 - GIY-YIG catalytic domain

PF02151 - UvrB/uvrC motif

FAMILY I.2.EndoV.COG1978.COG1515.KOG4417

gi|197622533|gb|EDY35104.1| Endonuclease V family [Aciduliprofundum boonei T469]

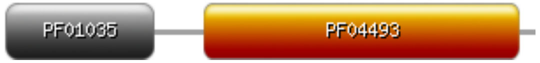

gi|56404428|sp|Q9X2H9.1|NFI_THEMA RecName: Full=Endonuclease V; AltName: Full=Deoxyinosine 3'endonuclease; AltName: Full=Deoxyribonuclease V; Short=DNase V

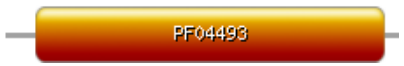

PF04493 - Endonuclease V

PF01035 - 6-O-methylguanine DNA methyltransferase, DNA binding domain

FAMILY D.21.PF01548.COG3547

gi|75440312|sp|Q6W1K1|Q6W1K1_RHISN Transposase

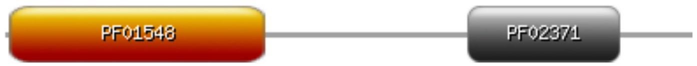

gi|194339287|gb|EDX20253.1| transposase [Streptomyces sp. Mg1]

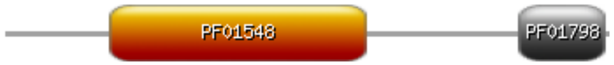

PF01548 - Transposase

PF02371 - Transposase IS116/IS110/IS902 family

PF01798 - Putative snoRNA binding domain

FAMILY D.20.PF01548.COG3547

gi|190410067|ref|YP_001965591.1| putative transposase protein [Sinorhizobium meliloti SM11]

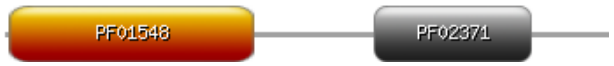

PF01548 - Transposase

PF02371 - Transposase IS116/IS110/IS902 family

FAMILY D.6.PF01548.COG3547

gi|116626083|ref|YP_828239.1| PF01548 transposase IS116/IS110/IS902 family protein [Candidatus Solibacter usitatus Ellin6076]

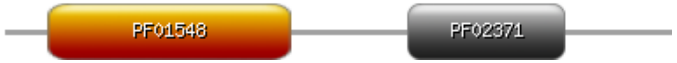

PF01548 - Transposase

PF02371 - Transposase IS116/IS110/IS902 family

FAMILY D.12.PF01548.COG3547

gi|189436097|gb|EDV05082.1| transposase, IS116/IS110/IS902 family [Bacteroides intestinalis DSM 17393]

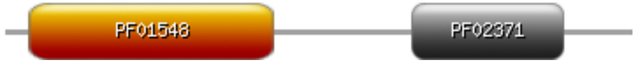

PF01548 - Transposase

PF02371 - Transposase IS116/IS110/IS902 family

FAMILY D.3.PF01548.COG3547

gi|130250|sp|P20665.1|PIV_MORBO PF01548 RecName: Full=Pilin gene-inverting protein; AltName: Full=PIVML

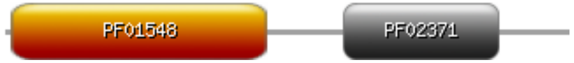

PF01548 - Transposase

PF02371 - Transposase IS116/IS110/IS902 family

FAMILY D.4.Terminase.PF03354.COG4626

gi|27805749|sp|P59217.1|TERL_BPSF5 RecName: Full=Putative terminase large subunit

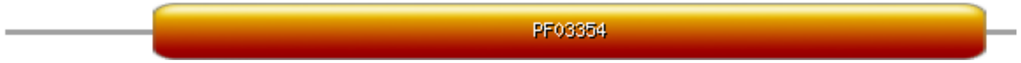

PF03354 - Phage Terminase

FAMILY D.17.Terminase.PF03237.PF04466

gi|375085720|ref|ZP_09732346.1| hypothetical protein HMPREF9454_00957 [Megamonas funiformis YIT 11815]

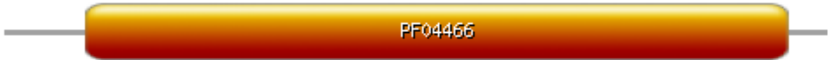

PF04466 - Phage terminase large subunit

FAMILY D.8.Terminase.PF03237.COG4373

gi|168213080|ref|ZP_02638705.1| PF03237 conserved hypothetical protein [Clostridium perfringens CPE str. F4969]

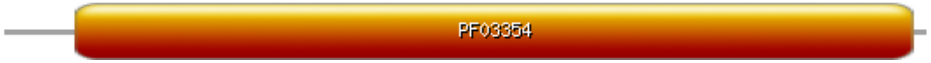

PF03354 - Phage Terminase

FAMILY D.5.Terminase.PF03237.PF04466.COG5362.COG5323

gi|288559543|ref|YP_003423029.1| PF03237 terminase large subunit [Methanobrevibacter ruminantium M1]

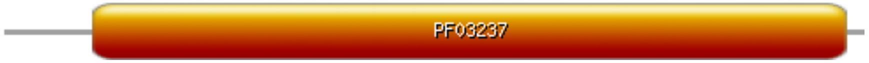

PF03237 - Terminase-like family

FAMILY D.23.Terminase.PF02499

gi|139645|sp|P16732.1|TRM3_HCMVA RecName: Full=Tripartite terminase subunit UL15 homolog; AltName: Full=DNA-packaging protein UL89; AltName: Full=Terminase large subunit

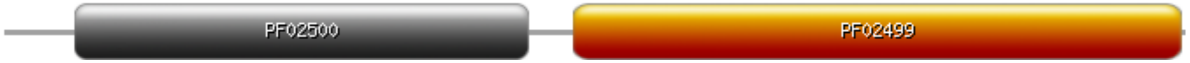

PF02499 - Probable DNA packing protein, C-terminus

PF02500 - Probable DNA packing protein, N-terminus

FAMILY D.16.Csn1.COG3513

gi|389815359|ref|ZP_10206685.1| CRISPR-associated protein, Csn1 family [Planococcus antarcticus DSM 14505]

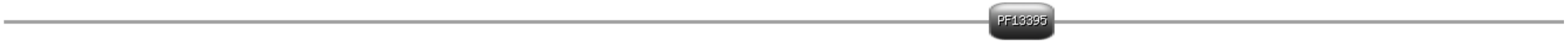

PF13395 - HNH endonuclease

FAMILY D.9.Spt6.COG2183.KOG1856

gi|210075180|ref|XP_002142995.1| YALI0B01224p [Yarrowia lipolytica]

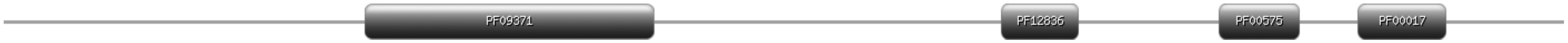

PF09371 - Tex-like protein N-terminal domain

PF12836 - Helix-hairpin-helix motif

PF00575 - S1 RNA binding domain

PF00017 - SH2 domain

FAMILY D.7.Tex.COG2183.KOG1857

gi|390940309|ref|YP_006404046.1| transcriptional accessory protein [Sulfurospirillum barnesii SES-3]

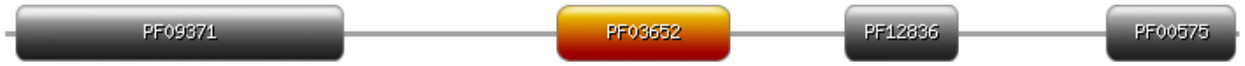

PF09371 - Tex-like protein N-terminal domain

PF12836 - Helix-hairpin-helix motif

PF00575 - S1 RNA binding domain

PF03652 - Uncharacterised protein family (UPF0081)

FAMILY D.10.RRXRR.PF14239

gi|124003405|ref|ZP_01688254.1| PF14239 paclitaxel/taxanoid biosynthesis susceptibility protein TS1 [Microscilla marina ATCC 23134]

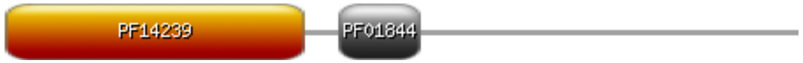

PF14239 - RRXRR protein

PF01844 - HNH endonuclease

FAMILY D.1.RuvX.YqgF.PF03652.COG0816

gi|335420460|ref|ZP_08551498.1| Holliday junction resolvase YqgF [Salinisphaera shabanensis E1L3A]

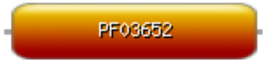

PF03652 - Uncharacterised protein family (UPF0081)

FAMILY D.24.RuvV.PF04848

gi|9629059|ref|NP_044078.1| PF04848 MC127R [Molluscum contagiosum virus subtype 1]

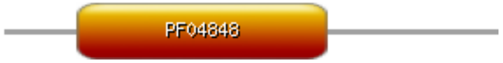

PF04848 - Poxvirus A22 protein

FAMILY D.13.Ydc2.PF09159

gi|241163695|ref|XP_002409328.1| conserved hypothetical protein [Ixodes scapularis]

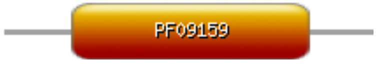

gi|1723534|sp|Q10423.1|CCE1_SCHPO RecName: Full=Cruciform cutting endonuclease 1, mitochondrial; AltName: Full=Protein ydc2; Flags: Precursor

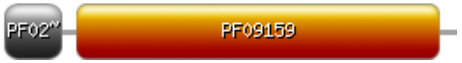

PF09159 - Mitochondrial resolvase Ydc2 / RNA splicing MRS1

PF02037 - SAP domain

FAMILY D.2.RuvC.PF02075.COG0817

gi|225849642|ref|YP_002729876.1| PF02075 crossover junction endodeoxyribonuclease RuvC [Persephonella marina EX-H1]

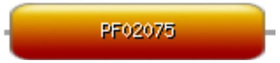

PF02075 - Crossover junction endodeoxyribonuclease RuvC

FAMILY D.18.DUF460.PF04312.COG2433

gi|302348960|ref|YP_003816598.1| hypothetical protein ASAC_1162 [Acidilobus saccharovorans 345-15]

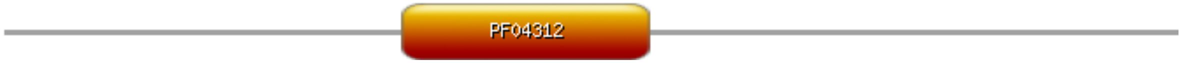

PF04312 - Protein of unknown function (DUF460)

FAMILY D.11.DUF429.PF04250.COG2410.COG4328

gi|345856455|ref|ZP_08808939.1| phosphoribosyl-ATP pyrophosphohydrolase domain protein [Desulfosporosinus sp. OT]

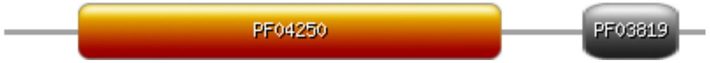

gi|353192388|gb|EHB57892.1| RelA/SpoT domain protein [Mycobacterium rhodesiae JS60]

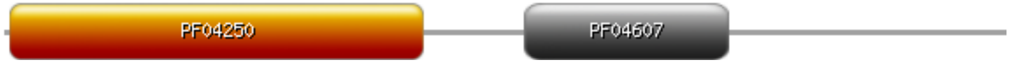

gi|188996797|ref|YP_001931048.1| hypothetical protein SY03AOP1_0866 [Sulfurihydrogenibium sp. YO3AOP1]

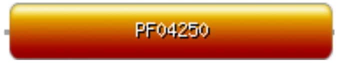

PF04250 - Protein of unknown function (DUF429)

PF03819 - MazG nucleotide pyrophosphohydrolase domain

PF04607 - Region found in RelA / SpoT proteins

FAMILY D.22.DUF429.PF04250

gi|262198305|ref|YP_003269514.1| hypothetical protein [Haliangium ochraceum DSM 14365]

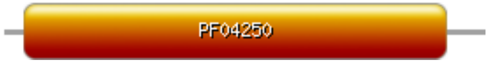

PF04250 - Protein of unknown function (DUF429)

FAMILY D.15.DUF429.PF04250.COG4923

gi|386846647|ref|YP_006264660.1| hypothetical protein ACPL_1695 [Actinoplanes sp. SE50/110]

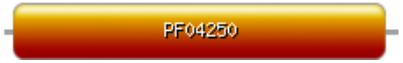

PF04250 - Protein of unknown function (DUF429)

FAMILY D.19.DUF3010.PF11215

gi|296271552|ref|YP_003654183.1| PF11215 hypothetical protein [Arcobacter nitrofigilis DSM 7299]

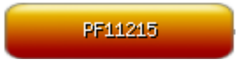

PF11215 - Protein of unknown function (DUF3010)

FAMILY D.14.PF03652

gi|385800816|ref|YP_005837220.1| resolvase RNase H domain protein fold protein [Halanaerobium praevalens DSM 2228]

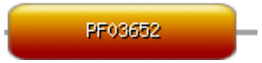

PF03652 - Uncharacterised protein family (UPF0081)

FAMILY D.25.DUF3882.PF07066

gi|9628684|ref|NP_043550.1| holin [Lactococcus phage c2]

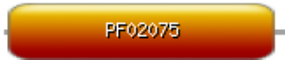

PF02075 - Crossover junction endodeoxyribonuclease RuvC

FAMILY II.1.DUF1280.PF06918

gi|390365579|ref|XP_794084.3| PREDICTED: uncharacterized protein LOC589347 [Strongylocentrotus purpuratus]

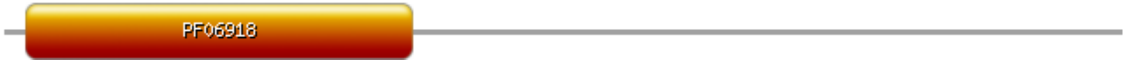

PF06918 - Protein of unknown function (DUF1280)

FAMILY II.2.RAG1.PF12940

gi|166092494|gb|ABY82262.1| recombination-activating protein 1 [Dinolestes lewini]

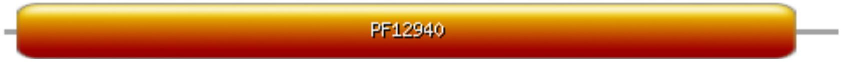

gi|131829|sp|P15919.1|RAG1_MOUSE RecName: Full=V(D)J recombination-activating protein 1; Short=RAG-1; Includes: RecName: Full=Endonuclease RAG1; Includes: RecName: Full=E3 ubiquitin-protein ligase RAG1

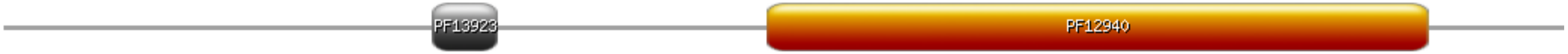

PF12940 - Recombination-activation protein 1 (RAG1)

PF13923 - Zinc finger, C3HC4 type (RING finger)

FAMILY E.7.RNH1.PF00075

gi|322702335|gb|EFY94003.1| PF00075 putative reverse transcriptase [Metarhizium anisopliae ARSEF 23]

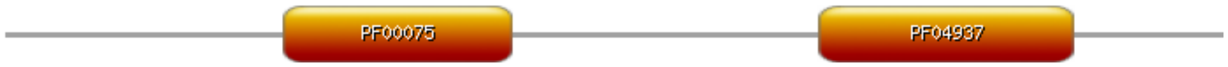

gi|321453153|gb|EFX64418.1| PF00075 hypothetical protein DAPPUDRAFT_266424 [Daphnia pulex]

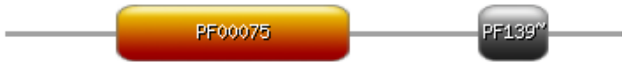

PF00075 - RNase H

PF04937 - Protein of unknown function (DUF 659)

PF13966 - zinc-binding in reverse transcriptase

FAMILY E.3.RNH1.PF00075.PF13456.COG0328.COG3341

gi|388490516|gb|AFK33324.1| unknown [Lotus japonicus]

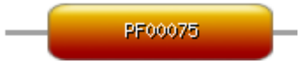

gi|81787696|sp|Q9KEI9.1|RNH1_BACHD RecName: Full=Ribonuclease H; Short=RNase H

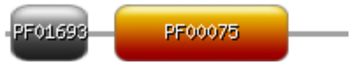

PF00075 - RNase H

PF01693 - Caulimovirus viroplasmin

FAMILY E.1.RNH1.PF00075.COG0328.COG3341.KOG3752

gi|339320311|ref|YP_004680006.1| ribonuclease H [Candidatus Midichloria mitochondrii IricVA]

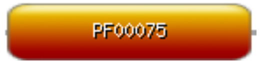

PF00075 - RNase H

FAMILY E.11.RNH1.PF00075

gi|52783154|sp|Q7SQ98.1|POL_ALV RecName: Full=Pol polyprotein; Contains: RecName: Full=Reverse transcriptase/ribonuclease H; Short=RT; Contains: RecName: Full=Integrase; Short=IN; AltName: Full=pp32

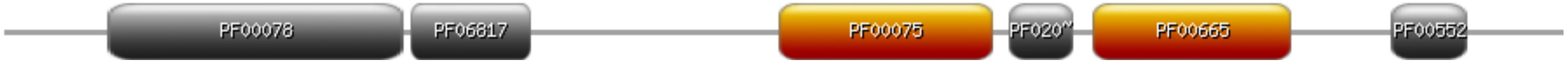

gi|52000846|sp|Q9UQG0.2|POK20_HUMAN RecName: Full=HERV-K_3q27.3 provirus ancestral Pol protein; Includes: RecName: Full=Reverse transcriptase; Short=RT; Includes: RecName: Full=Ribonuclease H; Short=RNase H; Includes: RecName: Full=Integrase; Short=IN

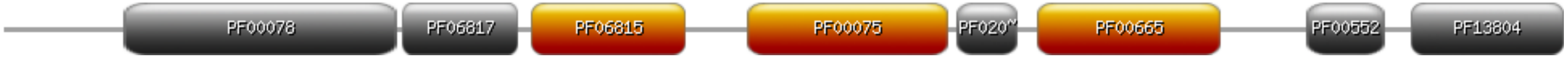

PF00665 - Integrase core domain

PF00078 - Reverse transcriptase (RNA-dependent DNA polymerase)

PF06817 - Reverse transcriptase thumb domain

PF00552 - Integrase DNA binding domain

PF02022 - Integrase Zinc binding domain

PF00075 - RNase H

PF13804 - Retro-transcribing viruses envelope glycoprotein

PF06815 - Reverse transcriptase connection domain

FAMILY E.8.RNH1.PF00075

gi|55740237|gb|AAV63810.1| PF00075 pol protein [Human immunodeficiency virus 1]

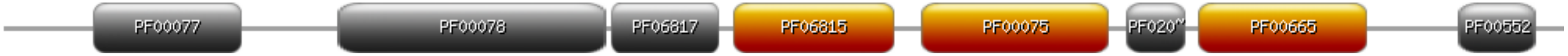

PF00077 - Retroviral aspartyl protease

PF00665 - Integrase core domain

PF06815 - Reverse transcriptase connection domain

PF00078 - Reverse transcriptase (RNA-dependent DNA polymerase)

PF00552 - Integrase DNA binding domain

PF06817 - Reverse transcriptase thumb domain

PF00075 - RNase H

PF02022 - Integrase Zinc binding domain

FAMILY E.4.RNH1.COG2801

gi|284434667|gb|ADB85376.1| putative retrotransposon protein [Phyllostachys edulis]

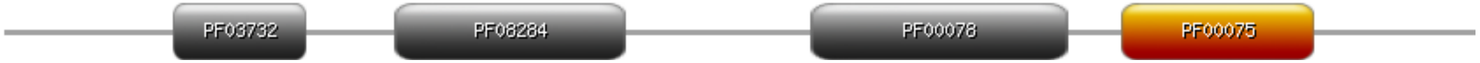

gi|130398|sp|P20825.1|POL2_DROME RecName: Full=Retrovirus-related Pol polyprotein from transposon 297; Includes: RecName: Full=Protease; Includes: RecName: Full=Reverse transcriptase; Includes: RecName: Full=Endonuclease

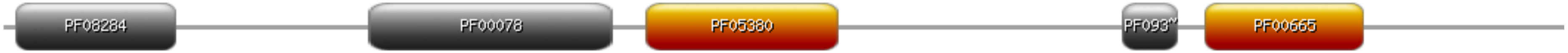

gi|384483425|gb|EIE75605.1| hypothetical protein RO3G_00309 [Rhizopus oryzae RA 99-880]

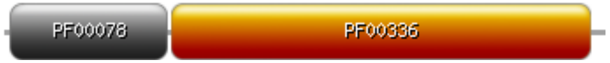

PF08284 - Retroviral aspartyl protease

PF00078 - Reverse transcriptase (RNA-dependent DNA polymerase)

PF03732 - Retrotransposon gag protein

PF00075 - RNase H

PF00665 - Integrase core domain

PF05380 - Pao retrotransposon peptidase

PF09337 - His(2)-Cys(2) zinc finger

PF00336 - DNA polymerase (viral) C-terminal domain

FAMILY E.9.YkuK.DUF458.PF04308.COG1978

gi|81342391|sp|O34776.1|YKUK_BACSU RecName: Full=Uncharacterized protein YkuK

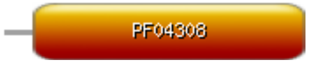

PF04308 - Protein of unknown function (DUF458)

FAMILY E.14

gi|307201692|gb|EFN81403.1| hypothetical protein EAI_09447 [Harpegnathos saltator]

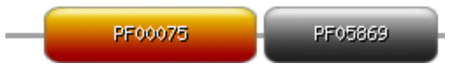

gi|327286446|ref|XP_003227941.1| PREDICTED: hypothetical protein LOC100566709 [Anolis carolinensis]

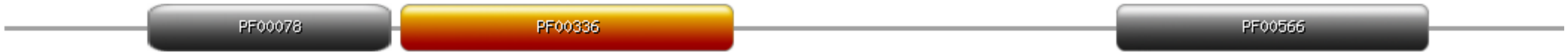

PF00075 - RNase H

PF05869 - DNA N-6-adenine-methyltransferase (Dam)

PF00566 - Rab-GTPase-TBC domain

PF00078 - Reverse transcriptase (RNA-dependent DNA polymerase)

PF00336 - DNA polymerase (viral) C-terminal domain

FAMILY E.5.RNH1.PF13456.PF00075

gi|147767745|emb|CAN76231.1| PF13456 hypothetical protein VITISV_040856 [Vitis vinifera]

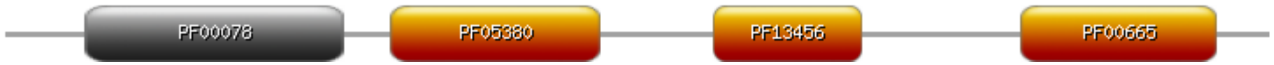

gi|75115132|sp|Q65WT8|Q65WT8_ORYSA Putative polyprotein

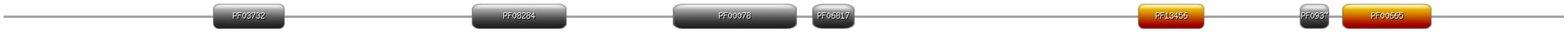

gi|14018045|gb|AAK52108.1|AC079936_4 PF13456 Putative retroelement [Oryza sativa Japonica Group]

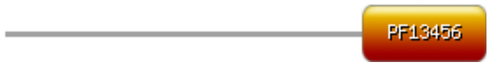

PF00665 - Integrase core domain

PF00078 - Reverse transcriptase (RNA-dependent DNA polymerase)

PF13456 - Reverse transcriptase-like

PF05380 - Pao retrotransposon peptidase

PF08284 - Retroviral aspartyl protease

PF03732 - Retrotransposon gag protein

PF09337 - His(2)-Cys(2) zinc finger

PF06817 - Reverse transcriptase thumb domain

FAMILY E.15.HepP.PF00336

gi|172046770|sp|P03158.2|DPOL_HBVA2 RecName: Full=Protein P; Includes: RecName: Full=DNA-directed DNA polymerase; Includes: RecName: Full=RNA-directed DNA polymerase; Includes: RecName: Full=Ribonuclease H

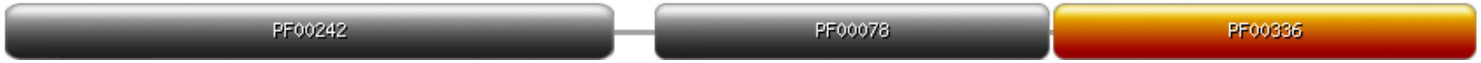

PF00242 - DNA polymerase (viral) N-terminal domain

PF00336 - DNA polymerase (viral) C-terminal domain

PF00078 - Reverse transcriptase (RNA-dependent DNA polymerase)

FAMILY E.10

gi|328700389|ref|XP_003241240.1| PREDICTED: hypothetical protein LOC100574845 [Acyrthosiphon pisum]

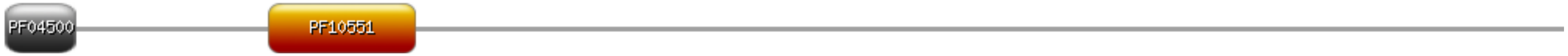

PF10551 - MULE transposase domain

PF04500 - FLYWCH zinc finger domain

FAMILY E.12

gi|221113337|ref|XP_002168602.1| PREDICTED: similar to predicted protein [Hydra magnipapillata]

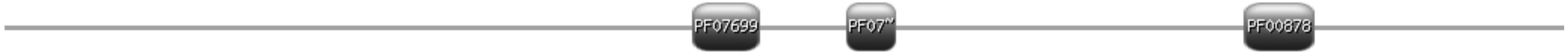

PF07699 - GCC2 and GCC3

PF00878 - Cation-independent mannose-6-phosphate receptor repeat

FAMILY E.2.RNH1.Ty1/Copia

gi|13124684|sp|P04146.3|COPIA_DROME RecName: Full=Copia protein; AltName: Full=Gag-int-pol protein; Contains: RecName: Full=Copia VLP protein; Contains: RecName: Full=Copia protease

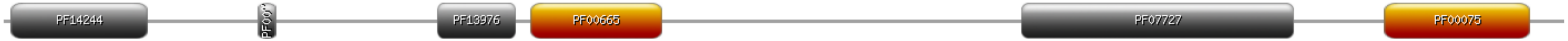

PF07727 - Reverse transcriptase (RNA-dependent DNA polymerase)

PF00665 - Integrase core domain

PF14244 - gag-polypeptide of LTR copia-type

PF13976 - GAG-pre-integrase domain

PF00075 - RNase H

PF00098 - Zinc knuckle

FAMILY E.6.Pao.PF05380

gi|254587304|emb|CAX83709.1| PF05380 Gag-Pol polyprotein [Schistosoma japonicum]

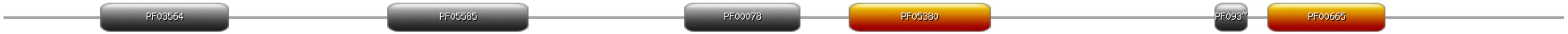

PF05380 - Pao retrotransposon peptidase

PF03564 - Protein of unknown function (DUF1759)

PF05585 - Putative peptidase (DUF1758)

PF00665 - Integrase core domain

PF00078 - Reverse transcriptase (RNA-dependent DNA polymerase)

PF09337 - His(2)-Cys(2) zinc finger

FAMILY E.16.COG5558

gi|257075532|ref|ZP_05569893.1| ISA1214-6 transposase [Ferroplasma acidarmanus fer1]

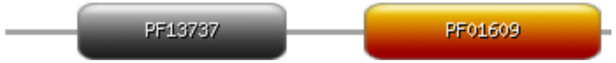

gi|11497921|ref|NP_069143.1| ISA1214-2 transposase [Archaeoglobus fulgidus DSM 4304]

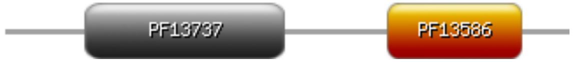

PF13737 - Transposase DDE domain

PF01609 - Transposase DDE domain

PF13586 - Transposase DDE domain

FAMILY III.4.Terminase.PF05876.COG5525

gi|313119126|gb|EFR42328.1| phage terminase large subunit (GpA) [Dialister microaerophilus UPII 345-E]

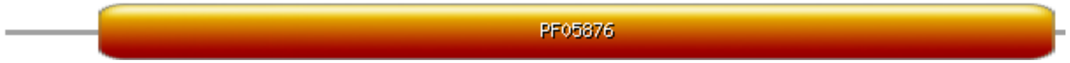

PF05876 - Phage terminase large subunit (GpA)

FAMILY III.2.Terminase.PF04466.PF03237.COG1783

gi|373124763|ref|ZP_09538602.1| PBSX family phage terminase, large subunit [Erysipelotrichaceae bacterium 21_3]

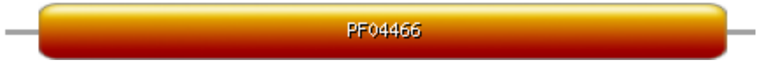

PF04466 - Phage terminase large subunit

FAMILY III.3.PF01526.COG4644

gi|338731831|ref|YP_004662950.1| hypothetical protein SNE_B24550 [Simkania negevensis Z]

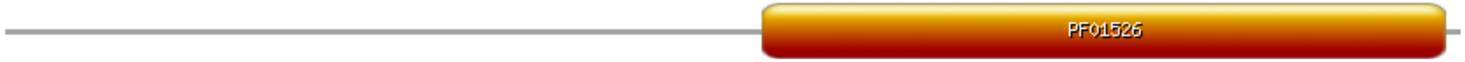

gi|54295894|ref|YP_122206.1| hypothetical protein plpp0051 [Legionella pneumophila str. Paris]

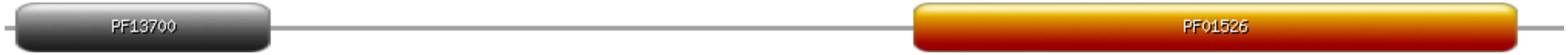

PF01526 - Tn3 transposase DDE domain

PF13700 - Domain of unknown function (DUF4158)

FAMILY III.1.DUF1258.PF02992.PF06869

gi|75326924|sp|Q7X7E7|Q7X7E7_ORYSA OSJNBa0027H06.6 protein

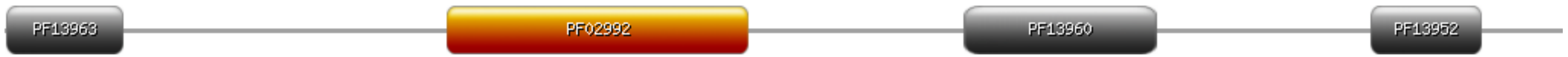

gi|147794504|emb|CAN71609.1| hypothetical protein VITISV_013246 [Vitis vinifera]

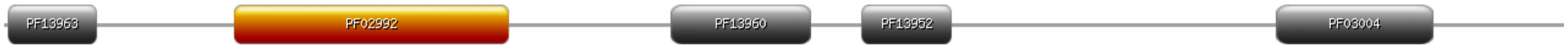

gi|356577576|ref|XP_003556900.1| PREDICTED: 22.7 kDa class IV heat shock protein-like [Glycine max]

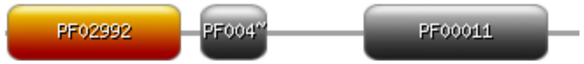

gi|122091425|tpe|CAK26778.2| TPA: transposase domain-containing protein [Tribolium castaneum]

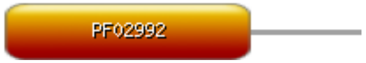

PF02992 - Transposase family tnp2

PF13960 - Domain of unknown function (DUF4218)

PF13952 - Domain of unknown function (DUF4216)

PF13963 - Transposase-associated domain

PF03004 - Plant transposase (Ptta/En/Spm family)

PF00011 - Hsp20/alpha crystallin family

PF00400 - WD domain, G-beta repeat

FAMILY IV.1.PF03050

gi|297555322|gb|EFH89186.1| RecB family nuclease, putative [Ktedonobacter racemifer DSM 44963]

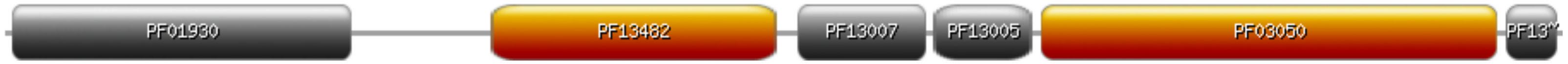

PF03050 - Transposase IS66 family

PF13482 - RNase_H superfamily

PF01930 - Domain of unknown function DUF83

PF13005 - putative Helix-turn-helix domain of transposase IS66

PF13007 - Transposase C of IS166 homeodomain

PF13817 - IS66 C-terminal element

FAMILY IV.2.PF03050

gi|381157003|ref|ZP_09866237.1| LOW QUALITY PROTEIN: Transposase IS66 family [Thiorhodovibrio sp. 970]

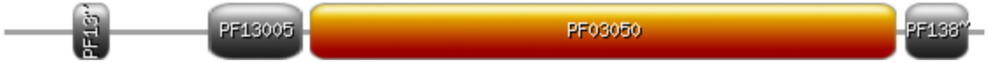

gi|2496673|sp|P55515.1|Y4JO_RHISN RecName: Full=Uncharacterized protein y4jO

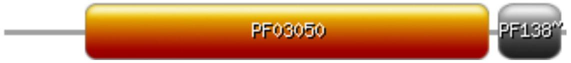

PF03050 - Transposase IS66 family

PF13817 - IS66 C-terminal element

PF13005 - putative Helix-turn-helix domain of transposase IS66

PF13007 - Transposase C of IS166 homeodomain

FAMILY IV.1.RNH2.PF01351.COG0164.COG1039.KOG2299

gi|313125351|ref|YP_004035615.1| PF01351 rnase hii [Halogeometricum borinquense DSM 11551]

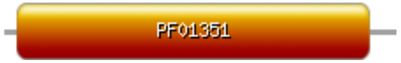

PF01351 - Ribonuclease HII

FAMILY IV.3.DUF4152.PF13680

gi|242399761|ref|YP_002995186.1| hypothetical protein TSIB_1786 [Thermococcus sibiricus MM 739]

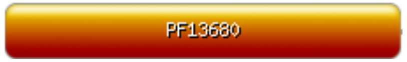

PF13680 - Protein of unknown function (DUF4152)

FAMILY V.1.DUF99.PF01949.COG1628

gi|24418790|sp|O28839.1|Y1433_ARCFU RecName: Full=UPF0215 protein AF_1433

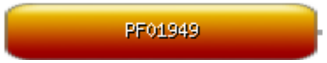

PF01949 - Protein of unknown function DUF99

FAMILY V.2.Prp8.PF12134.COG5178.KOG1795

gi|67460824|sp|Q6P2Q9.2|PRP8_HUMAN RecName: Full=Pre-mRNA-processing-splicing factor 8; AltName: Full=220 kDa U5 snRNP-specific protein; AltName: Full=PRP8 homolog; AltName: Full=Splicing factor Prp8; AltName: Full=p220

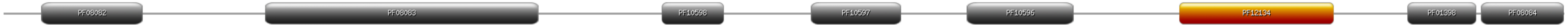

PF08083 - PROCN (NUC071) domain

PF12134 - PRP8 domain IV core

PF10596 - U6-snRNA interacting domain of PrP8

PF08082 - PRO8NT (NUC069), PrP8 N-terminal domain

PF10597 - U5-snRNA binding site 2 of PrP8

PF08084 - PROCT (NUC072) domain

PF10598 - RNA recognition motif of the spliceosomal PrP8

PF01398 - JAB1/Mov34/MPN/PAD-1 ubiquitin protease

FAMILY VI.1.Med13C.PF06333.KOG3600

gi|345560183|gb|EGX43308.1| hypothetical protein AOL_s00215g44 [Arthrobotrys oligospora ATCC 24927]

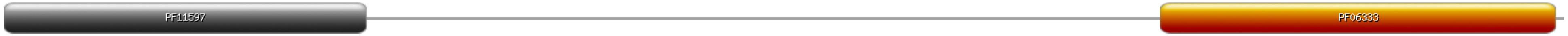

PF06333 - Mediator complex subunit 13 C-terminal

PF11597 - Mediator complex subunit 13 N-terminal

FAMILY VI.3.Maelstrom.PF13017

gi|229470209|sp|B4QL99.1|MAEL_DROSI RecName: Full=Protein maelstrom

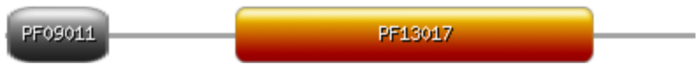

PF13017 - piRNA pathway germ-plasm component

PF09011 - Domain of unknown function (DUF1898)

FAMILY VI.2.Connect.PF06815

gi|55740237|gb|AAV63810.1| PF06815 pol protein [Human immunodeficiency virus 1]

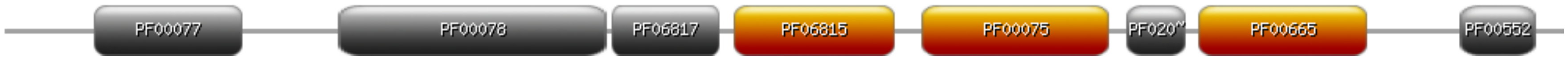

PF00077 - Retroviral aspartyl protease

PF06815 - Reverse transcriptase connection domain

PF00665 - Integrase core domain

PF00078 - Reverse transcriptase (RNA-dependent DNA polymerase)

PF00075 - RNase H

PF06817 - Reverse transcriptase thumb domain

PF00552 - Integrase DNA binding domain

PF02022 - Integrase Zinc binding domain

FAMILY F.25.PF01612

gi|308462810|ref|XP_003093685.1| hypothetical protein CRE_23111 [Caenorhabditis remanei]

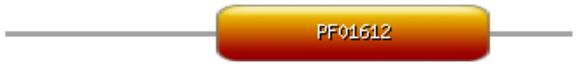

PF01612 - 3'-5' exonuclease

FAMILY F.16.PF01612.KOG2405

gi|241727922|ref|XP_002413785.1| PF01612 conserved hypothetical protein [Ixodes scapularis]

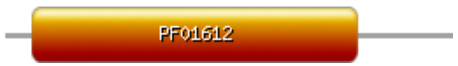

gi|301620462|ref|XP_002939597.1| PF01612 PREDICTED: LOW QUALITY PROTEIN: exonuclease 3'-5' domain-containing protein 1-like [Xenopus (Silurana) tropicalis]

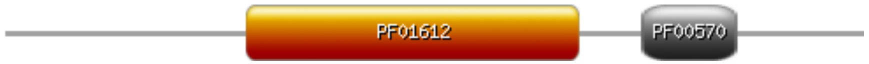

PF01612 - 3'-5' exonuclease

PF00570 - HRDC domain

FAMILY F.4.PF01612.COG0349.KOG2206

gi|91205505|ref|YP_537860.1| PF01612 rnd gene product [Rickettsia bellii RML369-C]

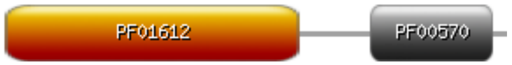

gi|14195186|sp|Q12149.1|RRP6_YEAST RecName: Full=Exosome complex exonuclease RRP6; AltName: Full=Ribosomal RNA-processing protein 6

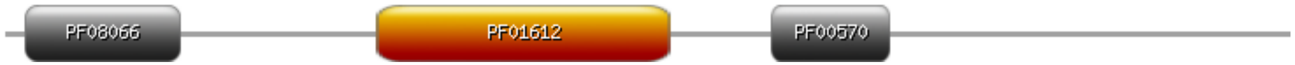

gi|300717019|ref|YP_003741822.1| PF01612 rnd gene product [Erwinia billingiae Eb661]

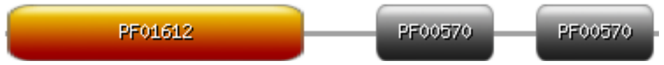

PF01612 - 3'-5' exonuclease

PF00570 - HRDC domain

PF08066 - PMC2NT (NUC016) domain

FAMILY F.11.PARN.PF04857.KOG1990

gi|71028506|ref|XP_763896.1| PF04857 hypothetical protein [Theileria parva strain Muguga]

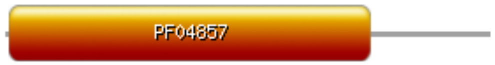

gi|60390262|sp|O95453.1|PARN_HUMAN RecName: Full=Poly(A)-specific ribonuclease PARN; AltName: Full=Deadenylating nuclease; AltName: Full=Deadenylation nuclease; AltName: Full=Polyadenylate-specific ribonuclease

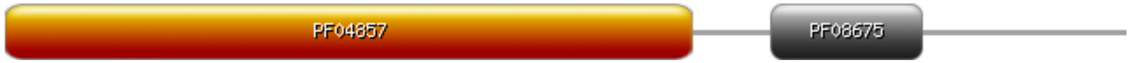

PF04857 - CAF1 family ribonuclease

PF08675 - RNA binding domain

FAMILY F.19.PF03175

gi|308476655|ref|XP_003100543.1| hypothetical protein CRE_19843 [Caenorhabditis remanei]

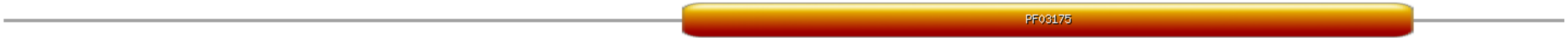

PF03175 - DNA polymerase type B, organellar and viral

FAMILY F.29.PF03175

gi|118849|sp|P03680.1|DPOL_BPPH2 RecName: Full=DNA polymerase; AltName: Full=Early protein GP2

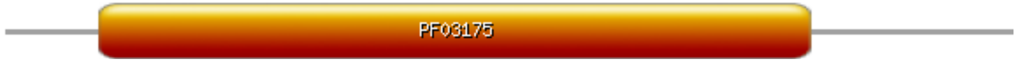

PF03175 - DNA polymerase type B, organellar and viral

FAMILY F.22.PF03175

gi|381214029|emb|CCE39359.1| PF03175 DNApol protein [Fowl adenovirus C]

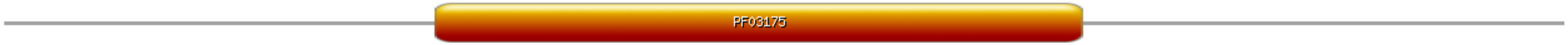

gi|2494187|sp|Q65946.1|DPOL_ADECC PF03175 RecName: Full=DNA polymerase

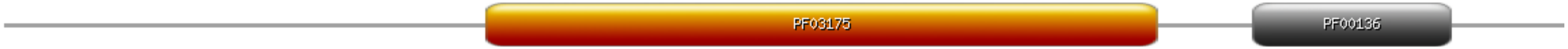

PF03175 - DNA polymerase type B, organellar and viral

PF00136 - DNA polymerase family B

FAMILY F.1.PF00929.PF13482.COG0847.COG2176.COG1199.KOG4793

gi|269215783|ref|ZP_06159637.1| PF00929 DNA polymerase III, alpha chain [Slackia exigua ATCC 700122]

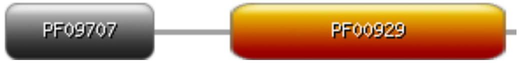

gi|14548235|sp|Q9HY82.1|RNT_PSEAE RecName: Full=Ribonuclease T; AltName: Full=Exoribonuclease T; Short=RNase T

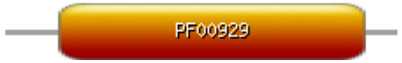

PF09707 - CRISPR-associated protein (Cas_Cas2CT1978)

PF00929 - Exonuclease

FAMILY F.23.PF13482

gi|75096643|sp|Q7Y2E1|Q7Y2E1_9CAUD Hypothetical protein

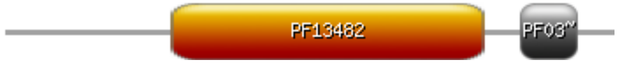

PF13482 - RNase_H superfamily

PF03811 - InsA N-terminal domain

FAMILY F.2.PF01612.COG0749

gi|383763177|ref|YP_005442159.1| polA gene product [Caldilinea aerophila DSM 14535 = NBRC 104270]

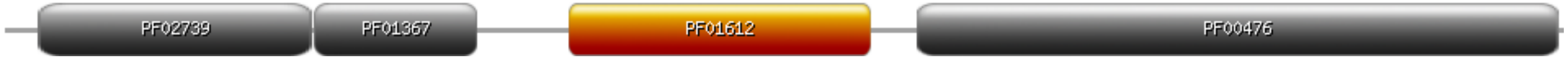

PF00476 - DNA polymerase family A

PF02739 - 5'-3' exonuclease, N-terminal resolvase-like domain

PF01367 - 5'-3' exonuclease, C-terminal SAM fold

PF01612 - 3'-5' exonuclease

FAMILY F.14.PF13482

gi|118856|sp|P00581.1|DPOL_BPT7 RecName: Full=DNA polymerase; AltName: Full=T7 DNA polymerase

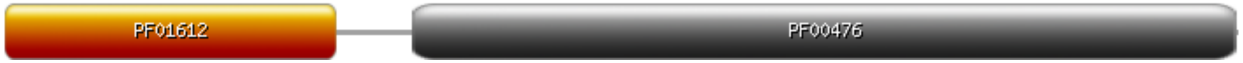

PF00476 - DNA polymerase family A

PF01612 - 3'-5' exonuclease

FAMILY F.13.PF13482.COG2251

gi|260061160|ref|YP_003194240.1| PF13482 hypothetical protein RB2501_06165 [Robiginitalea biformata HTCC2501]

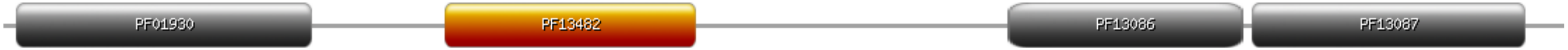

gi|16329373|ref|NP_440101.1| COG2251 hypothetical protein sll1686 [Synechocystis sp. PCC 6803]

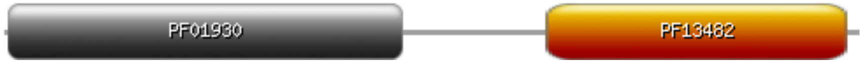

PF13087 - AAA domain

PF13482 - RNase_H superfamily

PF13086 - AAA domain

PF01930 - Domain of unknown function DUF83

FAMILY F.17.DUF2779.PF11074

gi|294339011|emb|CAZ87356.1| PF11074 conserved hypothetical protein [Thiomonas sp. 3As]

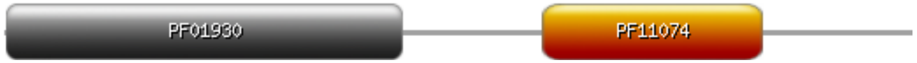

PF11074 - Domain of unknown function(DUF2779)

PF01930 - Domain of unknown function DUF83

FAMILY F.26.NSP11.PF06471

gi|383080785|ref|YP_005352870.1| orf1ab gene product [Wigeon coronavirus HKU20]

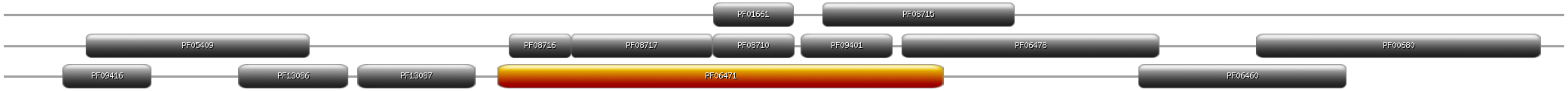

PF06471 - NSP11

PF06478 - Coronavirus RPol N-terminus

PF06460 - Coronavirus NSP13

PF05409 - Coronavirus

PF08717 - nsp8 replicase

PF09401 - RNA synthesis protein NSP10

PF08710 - nsp9 replicase

PF08715 - Papain like viral protease

PF08716 - nsp7 replicase

PF13087 - AAA domain

PF13086 - AAA domain

PF00680 - RNA dependent RNA polymerase

PF01661 - Macro domain

PF09416 - RNA helicase (UPF2 interacting domain)

FAMILY F.18.PF01612

gi|256617076|ref|ZP_05473922.1| DNA polymerase I [Enterococcus faecalis ATCC 4200]

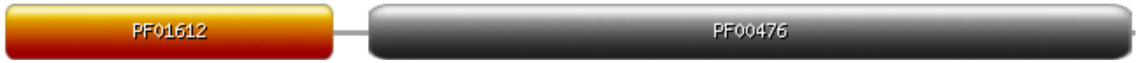

PF00476 - DNA polymerase family A

PF01612 - 3'-5' exonuclease

FAMILY F.27.PF09281

gi|118828|sp|P19821.1|DPO1_THEAQ RecName: Full=DNA polymerase I, thermostable; AltName: Full=Taq polymerase 1

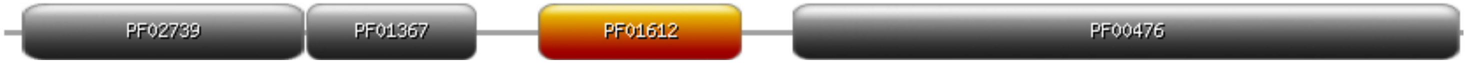

PF00476 - DNA polymerase family A

PF02739 - 5'-3' exonuclease, N-terminal resolvase-like domain

PF01367 - 5'-3' exonuclease, C-terminal SAM fold

PF01612 - 3'-5' exonuclease

FAMILY F.20.PF03175

gi|156398391|ref|XP_001638172.1| predicted protein [Nematostella vectensis]

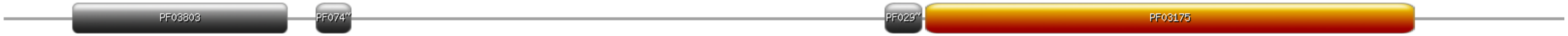

PF03803 - Scramblase

PF03175 - DNA polymerase type B, organellar and viral

PF07498 - Rho termination factor, N-terminal domain

PF02945 - Recombination endonuclease VII

FAMILY F.28.PF03337

gi|549293|sp|P36317.1|F12_FOWPN RecName: Full=Virion release protein; AltName: Full=Protein F12 homolog

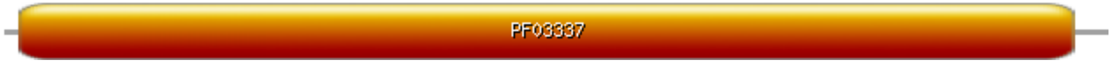

PF03337 - Poxvirus F12L protein

FAMILY F.6.PF01612.KOG4373.KOG2207

gi|334132717|ref|ZP_08506473.1| 3'-5' exonuclease [Methyloversatilis universalis FAM5]

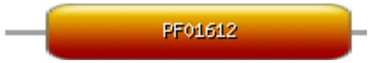

gi|6136393|sp|Q14191.1|WRN_HUMAN RecName: Full=Werner syndrome ATP-dependent helicase; AltName: Full=DNA helicase, RecQ-like type 3; Short=RecQ3; AltName: Full=Exonuclease WRN; AltName: Full=RecQ protein-like 2

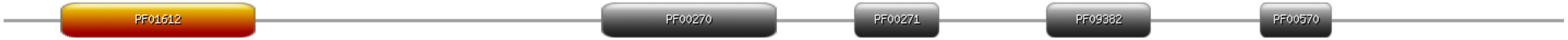

PF01612 - 3'-5' exonuclease

PF00270 - DEAD/DEAH box helicase

PF09382 - RQC domain

PF00271 - Helicase conserved C-terminal domain

PF00570 - HRDC domain

FAMILY F.9.Pop2.PF04857.COG5228.KOG0304

gi|145507628|ref|XP_001439769.1| PF04857 hypothetical protein [Paramecium tetraurelia strain d4-2]

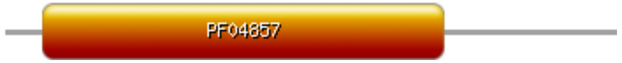

PF04857 - CAF1 family ribonuclease

FAMILY F.8.PF00929.COG5018.KOG0542

gi|302877099|ref|YP_003845732.1| PF00929 Exonuclease RNase T and DNA polymerase III [Clostridium cellulovorans 743B]

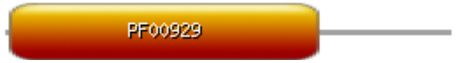

gi|254572319|ref|XP_002493269.1| PF00929 Protein of unknown function, rich in asparagine residues [Komagataella pastoris GS115]

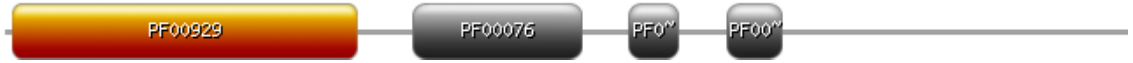

PF00929 - Exonuclease

PF00076 - RNA recognition motif. (a.k.a. RRM, RBD, or RNP domain)

PF00641 - Zn-finger in Ran binding protein and others

FAMILY F.3.PF03104.COG0417.KOG1798.KOG0970.KOG0969.KOG0968

gi|339756985|gb|EGQ40568.1| DNA polymerase, pol2 [Candidatus Nanosalinarum sp. J07AB56]

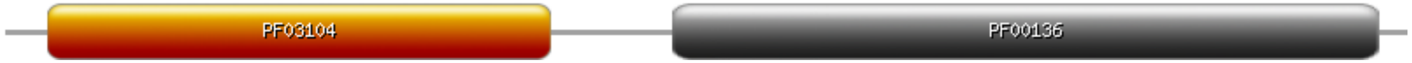

PF00136 - DNA polymerase family B

PF03104 - DNA polymerase family B, exonuclease domain

FAMILY F.10.PF13482.COG3359

gi|115377698|ref|ZP_01464891.1| PF13482 conserved hypothetical protein [Stigmatella aurantiaca DW4/3-1]

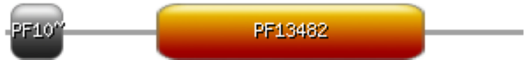

gi|15614328|ref|NP_242631.1| hypothetical protein BH1765 [Bacillus halodurans C-125]

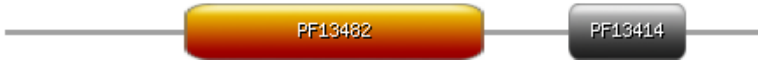

PF13482 - RNase_H superfamily

PF10391 - Fingers domain of DNA polymerase lambda

PF13414 - TPR repeat

FAMILY F.12.PF10108.COG3298

gi|325107365|ref|YP_004268433.1| PF10108 3'-5' exonuclease [Planctomyces brasiliensis DSM 5305]

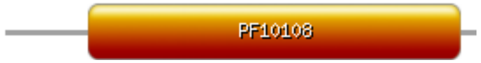

PF10108 - Predicted 3'-5' exonuclease related to the exonuclease domain of PolB

FAMILY F.15.ExoI.PF00929.COG2925

gi|124008030|ref|ZP_01692729.1| exodeoxyribonuclease I, putative [Microscilla marina ATCC 23134]

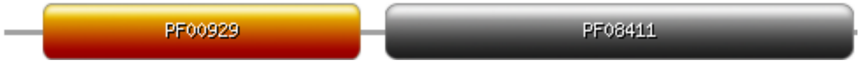

PF08411 - Exonuclease C-terminal

PF00929 - Exonuclease

FAMILY F.21.ExoVIII.PF00929

gi|78485014|ref|YP_390939.1| exonuclease [Thiomicrospira crunogena XCL-2]

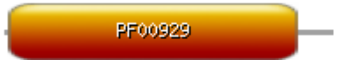

PF00929 - Exonuclease

FAMILY F.7.ORN.PF00929.COG1949.KOG3242

gi|1176352|sp|P45340.1|ORN_HAEIN RecName: Full=Oligoribonuclease

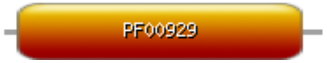

PF00929 - Exonuclease

FAMILY F.24.Ncap.PF00843

gi|81948384|sp|Q8BD30|Q8BD30_9VIRU Nucleocapsid protein

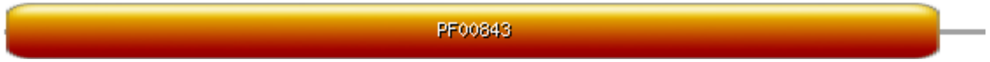

PF00843 - Arenavirus nucleocapsid protein

FAMILY Piwi.Argonaute.PF02171.COG1431.KOG1041.KOG1042

gi|74536972|sp|Q8U3D2|Q8U3D2_PYRFU Hypothetical protein PF0537

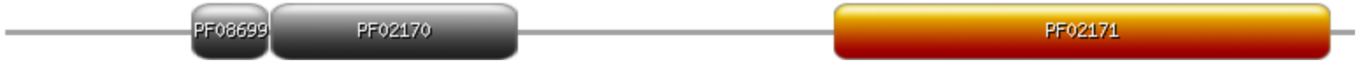

PF02171 - Piwi domain

PF02170 - PAZ domain

PF08699 - Domain of unknown function (DUF1785)

FAMILY Transpo.PF1385.COG0675

gi|257125268|ref|YP_003163382.1| PF01385 transposase, IS605 OrfB family [Leptotrichia buccalis C-1013-b]

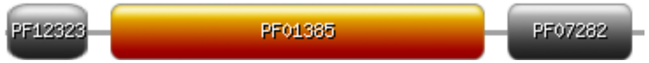

PF01385 - Probable transposase

PF07282 - Putative transposase DNA-binding domain

PF12323 - Helix-turn-helix domain
