# Supplementary material for: The RNase H-like superfamily: new members, comparative structural analysis and evolutionary classification
Source: Nucleic Acids Res. 2014 Jan 23;42(7):4160–79. doi: 10.1093/nar/gkt1414 (PMC3985635; doi:10.1093/nar/gkt1414)
Supplement: Supplementary Data [file supp_gkt1414_nar-02849-r-2013-File008.zip › List of Supplementary Files.docx]

**SUPPLEMENTARY DATA**

Supplementary File 1 – list of PDB identifiers for protein structures used at different steps of the RNHL superfamily analysis.

Supplementary File 2 – A zipped archive of FASTA files with all non-redundant RNHL sequences analyzed in this work (60923 sequences assigned to clusters and 1000 sequences not assigned to any cluster).

Supplementary File 3 – sequence alignment of a conserved RNHL core in 304 representative sequences (two representatives per family analyzed in this work), including 41 sequences with known structures.

Supplementary File 4 – an input file for MrBayes calculations, including a trimmed version of the alignment shown in Supplementary Figure 3

Supplementary File 5 – a phylogenetic tree in the Newick format, calculated based on the input from Supplementary File 4

Supplementary File 6 – a phylogenetic tree in the Newick format, calculated based on the input from Supplementary File 4 in which non-sequence data was excluded.

Supplementary File 7 – a phylogenetic tree in the Newick format, calculated based on the input from Supplementary File 4 in which multiple sequence alignment was excluded.

Supplementary File 8 – a phylogenetic tree in the Newick format, calculated based on the input from Supplementary File 4 in which catalytic residues conservation scores were excluded.

Supplementary File 9 – a phylogenetic tree in the Newick format, calculated based on the input from Supplementary File 4 in which pairwise family similarity scores were excluded.

Supplementary File 10 – a phylogenetic tree in the Newick format, calculated based on the input from Supplementary File 4 in which weighting of non-sequence data was reversed.

Supplementary Table 1 – Description of RNHL clusters, with operational names used in this work, COG, KOG, and Pfam family identifiers, and selected known members indicated. Known or predicted endo- or 3ʹ-5ʹ exonuclease function of individual families is also assigned: ‘1’ indicates that members of the family have known endo- or exonucleases activity or we expect it based on the similarity to other proteins. ‘?’ indicates the lack of activity or the lack of reliable predictions.

Supplementary Table 2 – Meta-BASIC pair-wise scores between newly detected and known RNHL families catalogued in the PFAM database. The highest score between each new RNHL family and its closest known homolog are shown in bold.

Supplementary Figure 1 – graphical representation of typical domain compositions of members of individual RNHL families
