# Supplementary material for: The RNase H-like superfamily: new members, comparative structural analysis and evolutionary classification
Source: Nucleic Acids Res. 2014 Jan 23;42(7):4160–79. doi: 10.1093/nar/gkt1414 (PMC3985635; doi:10.1093/nar/gkt1414)
Supplement: Supplementary Data [file supp_gkt1414_nar-02849-r-2013-File008.zip › SupplementaryFile1.doc]

**List 1: PDB IDs of protein structures representing RNHL SCOP families, used as queries to search the Protein Data Bank (PDB) with DALI:**

1eke, 2rn2, 1hjr, 1bco, 1b7e, 1qs4, 1u04, 1noy, 1uoc, 1vhx, 2kfn, 1kcf, 1vrt, 3bzk, 3e9l, 1zup, 2bw3

**List 2: PDB IDs of structures selected from DALI results, based on sequence similarity clustering, and used as queries in sequence database searches:**

1eke, 2rn2, 1hjr, 1bco, 1b7e, 1qs4, 1u04, 1noy, 1uoc, 1vhx, 2kfn, 1kcf, 1s1x, 2g8h, 2nrt, 1q8i, 1vk0, 1fxx, 1y97, 1w0h, 1w9h, 2f96, 1j9a, 2a1r, 2bw3, 1xhx, 2bdp, 2ktq, 2fbt, 2hbj, 1vsd, 1iv0, 2f7t, 1tk5, 1wlj, 1j54, 2d0a, 2hb5, 3bzk, 3e9l, 1zup, 2w36, 2qh9, 3n4q, 3psi, 3cpe, 1taq, 2wbn, 3tai, 1mu2, 3mwp

**List 3: PDB IDs of the 41 representative structures of the RNHL superfamily, whose comparison was used to guide manual adjustments of sequence alignment:**

1eke, 2rn2, 1hjr, 1bco, 1b7e, 1qs4, 1u04, 1uoc, 1vhx, 2kfn, 1kcf, 1s1x, 2g8h, 2nrt, 1q8i, 1w0h,1fxx, 2f96, 1j9a, 2a1r, 2bw3, 1xhx, 2fbt, 2hbj, 2f7t, 1tk5, 1wlj, 3bzk, 3e9l, 1zup, 2w36, 2qh9, 3n4q, 3psi, 3cpe, 1taq, 2wbn, 3tai, 1mu2, 3mwp, and unpublished coordinates of protein PA2046 obtained from the authors (Zhi-Jie Liu, personal communication)
